# Distribution pattern and health risk assessment of polycyclic aromatic hydrocarbons in the water and sediment of Algoa Bay, South Africa

**DOI:** 10.1007/s10653-018-0213-x

**Published:** 2018-11-11

**Authors:** A. O. Adeniji, O. O. Okoh, A. I. Okoh

**Affiliations:** 1 SAMRC Microbial Water Quality Monitoring Centre, University of Fort Hare, Alice, 5700 South Africa; 2 Department of Chemistry, University of Fort Hare, Alice, 5700 South Africa; 3 Applied and Environmental Microbiology Research Group, Department of Biochemistry and Microbiology, University of Fort Hare, Alice, 5700 South Africa

**Keywords:** Algoa bay, Polycyclic aromatic hydrocarbons, Human health risk assessment, Diagnostic ratios, Mutagenic equivalent, Marine environment

## Abstract

Polycyclic aromatic hydrocarbons are amongst the pollutants of major concern in the terrestrial and aquatic habitats. They are mostly characterised by carcinogenic and non-carcinogenic effects. Distribution and potential health risks of sixteen priority PAHs in the water and sediment samples collected between December 2015 and June 2016 from Algoa Bay, South Africa, were evaluated. Water and sediment samples collected were extracted with liquid–liquid and soxhlet extraction methods, respectively, and then cleaned up using glass column loaded with silica gel. Final concentrations of the target PAHs were determined by gas chromatography interfaced with flame ionization detector. Results indicated that individual PAH concentrations in surface water, bottom water and sediment samples ranged from not detected (ND) to 24.66 µg/L, ND to 22.81 µg/L and ND to 5.23 mg/kg correspondingly. Total PAHs concentrations varied as 12.78–78.94 µg/L, 1.20–90.51 µg/L and 1.17–10.47 mg/kg in the three environmental matrices in that order. The non-carcinogenic risk was generally below 1, whereas risk indices (dermal contact) were above the acceptable limit of 1 × 10^−4^ in the water column, suggesting possible carcinogenic effects to humans, with adults being the most vulnerable. Similarly, highest contributions to TEQs and MEQs in the sediments were made by benzo(a)pyrene and dibenzo(a,h)anthracene, the two most toxic congeners, signifying the possibility of carcinogenicity and mutagenicity in humans. Diagnostic ratios of PAHs reflect a prevailing pyrogenic input all through. The pollution was albeit moderate, yet regular check is recommended to ensure safe and healthy environment for human and aquatic lives.

**Electronic supplementary material:**

The online version of this article (10.1007/s10653-018-0213-x) contains supplementary material, which is available to authorized users.

## Introduction

Polycyclic aromatic hydrocarbons (PAHs) are a group of dangerous organic substances containing two or more fused benzene rings which are found everywhere in the environment (Huggett et al. 1992; Pavlova and Ivanova 2003). More than 200 fractions of PAHs have been identified. They are primarily classified as low molecular and high molecular PAHs. Lower class is constituted by members with two or three fused aromatic rings, while the higher class is composed of those with a minimum of four benzene rings and is more stable in the environment. PAHs are naturally hydrophobic, lipophilic and exhibit great tendency of adsorption to suspended particulates in the aquatic systems. They are commonly deposited in the bottom sediments, thus accumulating to the levels higher enough to exercise toxic effects upon the environment (Prabhukumar and Pagilla 2010; Brazkova et al. 2013; Olatunji et al. 2014). PAHs are also bio-available to the aquatic animals and consequently find their ways into the dietary sources (Sakuma et al. 2011). They are assumed to have potentials for endocrine system disruption (Wilson et al. 2001) and are also listed as priority organic pollutants with their photo-oxidation products and alkylated derivatives on account of their tendency to be carcinogenic, teratogenic and/or mutagenic (Sakuma et al. 2011).

These aromatic compounds are naturally present in the fossil fuels and find their ways into the environment as by-products of incomplete combustion of organic materials (e.g. oil and gas, coal, biomass, fire wood, garbage, tobacco or charbroiled meat) by way of incineration, vehicular exhaust emissions, oil exploration, power generation and various industrial production practices (Pavlova and Ivanova 2003; Cai et al. 2009; Fang et al. 2010; Jamhari et al. 2014). Larger quantity of these hazardous compounds arrive the marine environments from coastal region as urban run-off, domestic wastes, river run-off, industrial discharges and also from vessels as ballast waters, emissions from engine and bilge pumping. They enter the aquatic bodies at times as leachates from bulwarks and docksides (Jamhari et al. 2014; Kennish 1994; Irwin et al. 1997.

Algoa Bay is an important coastal resource in South Africa for its marine biodiversity and the largest amongst the bays facing eastward along the south coast of the country. It is found in the Nelson Mandela Bay Metropolitan Municipality of the Eastern Cape Province, South Africa with great recreational and socio-economical values. Recreational activities in the area include fishing, sailing, swimming, water sporting, and pleasure cruises amongst others (Klages and Bornman 2005; Klages et al. 2011; AES 1999). It is a habitation, occasional breeding and nursing ground for many marine mammals, fish and birds, and some other invertebrates (AES 1999; SST 2006; BirdLife International 2009; Fourie 2013; Bottomley 2014). The bay is shallow with roughly 30 m depths across all its catchment area and receives fairly large influx of wastes from Swartkops and Sundays Rivers (AES 1999; CSIR 2007).

Klages and Bornman (2003, 2005) carried out a brief investigation of the levels of oil and grease, polycyclic aromatic hydrocarbons (PAHs) and total petroleum hydrocarbons as pollutants in the surface water (1 m depth) of Algoa Bay more than a decade ago. PAHs at the time were detected in higher concentrations around St. Croix Island (Bornman 2003), even though the levels were fluctuating over time. However, the studies were restricted to the surface water alone (Klages and Bornman 2003, 2005). Till date, no other pollution study (especially on hydrocarbons) has been documented in the study area to the best of our knowledge, except the evaluation of the TPH levels in the surface and bottom waters, as well as the sediment that were reported recently by Adeniji et al. (2017). The aim of this research work was therefore to assess the levels of the 16 priority PAHs at the two water depths (surface and bottom) and also in the sediment of Algoa Bay. Possible level of health risk to humans was also determined using US EPA Hazard Quotient Risk Calculation model RISC 4.02, and likely sources of these environmental pollutants were identified using some key isomeric ratios.

## Materials and methods

### Description of the study area

**Fig. 1:**
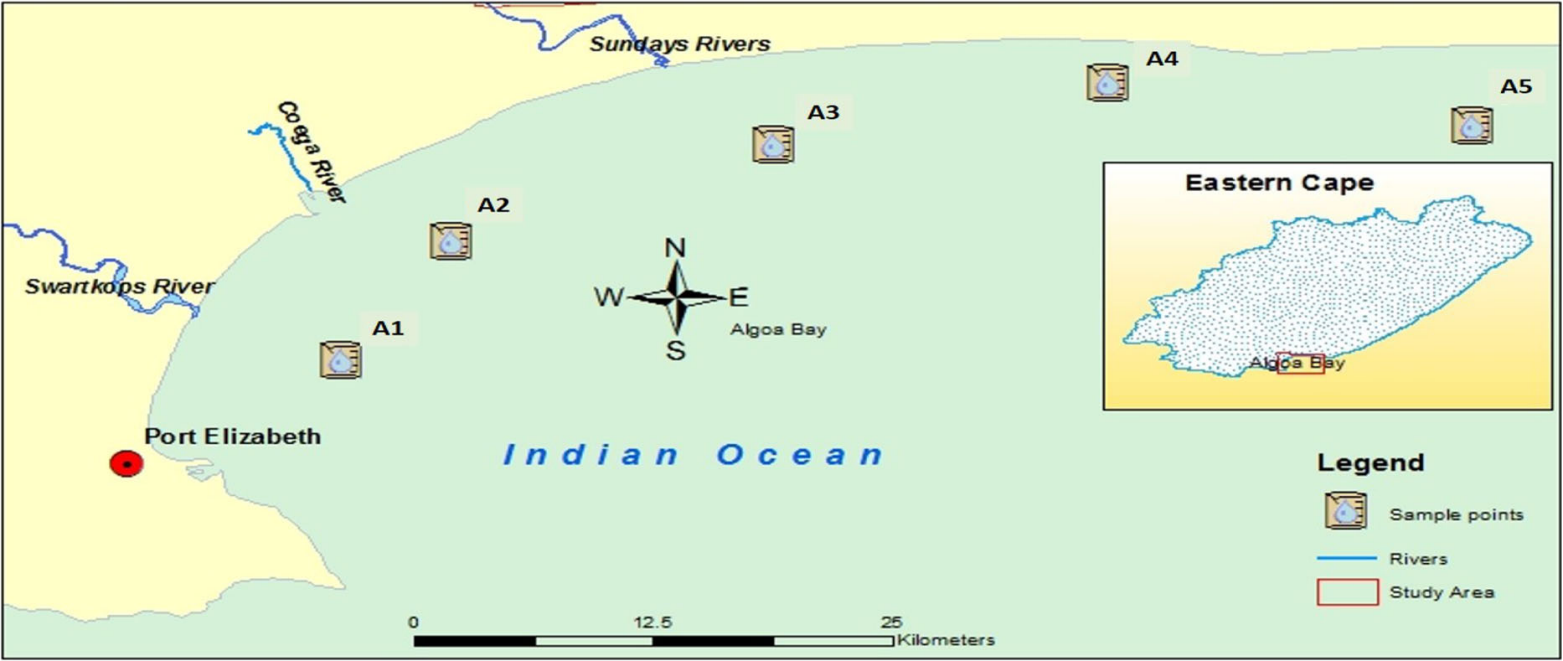
Map of Algoa Bay

Algoa Bay (latitude: 33.83°S and longitude: 25.80°E) is situated in the Nelson Mandela Bay Metropolitan Municipality, close to Port Elizabeth city and Coega deep water port facility in Eastern Cape, South Africa. It is about 683 kilometres east of the Cape of Good Hope and welcomes organic nutrients and untreated waste waters from its four estuaries (SST 2006; CSIR 2007). Features of the five sampling locations are presented in the Fig. 1 and Table 1. Other details are available in the previous report by Adeniji et al. (2017).

**Table 1:** Features of the study area

Study location	Sampling points	Latitude	Longitude	Description
Algoa Bay	1	33.9°S	25.70°E	Sheltered Bay
	2	33.83°S	25.75°E	St Croix
	3	33.77°S	25.91°E	Sundays Estuary
	4	33.73°S	26.06°E	Alexandria Dune Fields
	5	33.76°S	26.24°E	Woody Cape

### Sources of solvents, reagents and standards

HPLC grade solvents, anhydrous sodium sulphate (AR grade) and silica gel (100–200 mesh) used for the extraction and cleanup were sourced from Merck, Germany, while n-pentane (AR grade) was acquired from Avantor Performance Materials, Poland and concentrated hydrochloric acid from Radchem Laboratory Supplies, South Africa.

Surrogate standards (o-terphenyl, 2-fluorobiphenyl) and calibration standards of the 16 priority PAHs, containing 2000 µg/mL each of the components: [naphthalene (Nap), acenaphthene (Ace), acenaphthylene (Acy), fluorene (Flu), phenanthrene (Phe), anthracene (Ant), fluoranthene (Flt), pyrene (Pyr), benzo[a]anthracene (BaA), chrysene (Chr), benzo[b]fluoranthene (BbF), benzo[k]fluoranthene (BkF), benzo(a)pyrene (BaP), dibenzo[a,h]anthracene (DiahA), indeno[1,2,3-cd]pyrene (InPy) and benzo[g,h,i]perylene (BghiP)] combined as a mixture were ordered from Restek and Accustandards (USA).

### Sample collection, extraction and cleanup

All glass bottles and vials used in this work were initially soaked in 10% nitric acid before they were washed with soap and rinsed successively with tap water, double-distilled water and acetone. The glass wares were subsequently drained and dried in an oven at 105 °C for about 12 h. PTFE lined lids were used in covering all (Adeniji et al. 2017; Salaudeen et al. 2018). Fifty water samples each from the surface (10 cm depth) and bottom levels (30 m depth), and twenty-four sediment samples were taken in the morning each time between 7 am and 10 am from five sampling locations in Algoa Bay between December, 2015 and June, 2016. SeaBird 19plus V2 CTD SBE 55 Carousel with six 4L niskin bottles was used in the collection of water samples that were immediately transferred into pre-cleaned amber glass bottles, whereas sediment samples were taken using stainless steel cone dredge. The water samples were adjusted to pH < 2 using 6 M concentrated hydrochloric acid, and they were immediately moved to the laboratory on ice chest at temperature below 4 °C for chemical analysis (Kafilzadeh et al. 2011; Gorleku et al. 2014).

A measure of the water sample (500 mL each) was spiked with surrogate standard mixture containing 10 µg/mL each of o-terphenyl and 2-fluorobiphenyl (bought from Restek, USA) and extracted three times with 20 mL portion of n-hexane each time in a separatory funnel. The organic layers were pooled together after being filtered through anhydrous sodium sulphate (to remove traces of water) and subsequently concentrated to about 2 mL in a rotary evaporator (IKA RV8, Shanghai China). Sediment samples were air-dried in the dark for 5 days, crushed and sieved with 0.5 mm mesh. About 10 g aliquot of the crushed sediment sample was mixed with adequate quantity of anhydrous sodium sulphate to make a flowing powder. Each sample was spiked with mixture of two surrogate standards as earlier mentioned and extracted using 200 mL of dichloromethane for 24 h in a Soxhlet extraction apparatus. The extract was allowed to settle, filtered through anhydrous sodium sulphate in a funnel and afterwards concentrated to about 2 mL using a rotary evaporator. The concentrate was thereafter solvent-exchanged to n-hexane before column cleanup (Kafilzadeh et al. 2011; WSDE 1997; Ogunfowokan et al. 2003; Jiao et al. 2009).

The concentrated extracts were subjected to column chromatography for the elimination of certain co-extracted compounds that could possibly bring about interference during instrumental analysis of the target compounds. Each of the water and sediment extracts was made to pass through a chromatographic column (10 mm I.D. × 30 cm) plugged with glass wool and packed with a slurry of 10 g of activated silica gel (100–200 mesh) in dichloromethane with a layer of 2 cm anhydrous sodium sulphate on top. Pre-conditioning of the column was done using 20 mL of n-hexane before the sample extract was introduced. The amber glass vial containing the extract was thereafter rinsed with about 2 mL of n-hexane into the column, and the first elution was achieved with 20 mL of n-pentane. This fraction was set aside for aliphatic hydrocarbons analysis. Final elution was, however, done using 40 mL of dichloromethane: n-pentane (2:3 v/v), and the eluate was collected and kept for aromatic hydrocarbons determination. This second fraction was eventually concentrated to ~ 2 mL, solvent-exchanged to dichloromethane, finally reduced to ~ 1 mL and stored in amber glass vial in the refrigerator at below 4 °C for gas chromatographic determination (Jamhari et al. 2014; WSDE 1997; KDHE 2015; Benson et al. 2017).

The moisture content of the sediment samples was gravimetrically determined as described by Motsara and Roy (2008). Loss of weight on ignition was used to estimate the levels of the organic carbon (OC) and organic matter (OM) contents in sediments (Klages and Bornman 2005; Motsara and Roy 2008; Olutona et al. 2016).

### Gas chromatography analysis of the 16 polycyclic aromatic hydrocarbons

The PAHs levels in the concentrated extracts were determined using Agilent 7820A gas chromatograph (GC) coupled with flame ionization detector (Olatunji et al. 2014; Coelho et al. 2008; Essumang 2010; Nekhavhambe et al. 2014; Omores et al. 2017). A HP-5 fused silica capillary column (30 m × 0.320 mm i.d. and 0.250 μm film thickness) was used with helium (99.999%) as carrier gas at a constant flow rate of 1.63 mL/min. Splitless injection of 1 μL of the extract was made via an autosampler. The oven temperature of the instrument was programmed to start from 70 °C (held for 3 min), then increased to 325 °C at 12 °C/min and held for 6 min. The detector was operated at 300 °C with H_2_, N_2_ and air flow rates of 45.0 mL/min, 20 mL/min and 450 mL/min, respectively (Kafilzadeh et al. 2011; Nekhavhambe et al. 2014; Hussein et al. 2016).

Each analyte peak was identified using retention time of the corresponding PAH standard while the peak area was determined using the baseline–baseline mode of the Agilent Chemstation software. Working standards (100 µg/mL) were prepared by diluting the stock standards with n-hexane. Set of standards used for calibration were then prepared from these by serial dilution in the working range of 0.05–20 μg/mL (Olatunji et al. 2014). Calibration curves were plotted for all the 16 PAHs and verified from time to time with a middle level calibration standard. Linearity of the curves ranged from 0.9974 to 0.9990 and were all within the acceptable range of *r*^2^ ≥ 0.990 (Cortes et al. 2012; Yahaya et al. 2017). Average response factor was generated for each congener using the software and concentrations of PAHs in the sample extracts were estimated based on the response factors (Nekhavhambe et al. 2014; Ma et al. 2010).

Sample analysis was performed in duplicate, and average concentrations were reported. Middle level calibration and surrogate standards (2-fluorobiphenyl and o-terphenyl) were spiked into the samples to determine the method performance and matrix effects, respectively. The surrogate standard recoveries fell within the standard range and were used to make necessary corrections to the concentrations of the analytes determined in the study. The mean recovery levels of 79.53 ± 10.59% and 72.20 ± 9.82%, which were within the acceptable range recommended by US EPA, were obtained for the water and sediment samples correspondingly (KDHE 2015; ESS Laboratory 2008; Mirza et al. 2012).

Method blanks were also analysed alongside each batch of fifty samples using double-distilled water. An intermediate level solution containing all the compounds of interest (5 mg/L) was injected eight times into the instrument, and standard deviations of their respective areas were calculated. Mean values obtained were divided by standard deviations of the replicates to determine the signal-to-noise ratios (S/N), which were all higher than 5 as required (WDNR 1996). Hence, limit of detection (LOD) and limit of quantification (LOQ) were subsequently estimated as a product of student *t* value at 99% confidence level for the eight injections and three and ten multiples of the standard deviation, respectively (Coelho et al. 2008; Schwarz et al. 2004; USP 2007; Kumar et al. 2015. Method blanks were all below the limits of detection, as the estimated LOD and LOQ for the 16 PAHs varied as 0.01–0.03 μg/L and 0.04–0.15 μg/L correspondingly. In calculation, all sample concentrations lesser than the detection limits were considered to be zero but reported as not detected (ND). The method’s precision estimated as the percentage ratio of the standard deviation of the eight replicate injections to the mean value was generally below 10 as necessary (Olatunji et al. 2014; Gorleku et al. 2014; Coelho et al. 2008; Kumar et al. 2015; US EPA 2003; Wei et al. 2015).

### Health risk assessment to humans

Assessment of health risk to humans is achieved by calculating the possibility of any severe health effects coming from the exposure of an individual to carcinogenic and/or non-carcinogenic substances over a particular period of time (Gerba 2006; Kamunda et al. 2016; US EPA 2001; US EPA 2009). In this study, health risks that may arise when an individual or a population is exposed to the 16 priority PAHs in the water and sediment of Algoa Bay was estimated using the US EPA standard models (US EPA 1989, 2004; ATSDR 2005).

### Non-carcinogenic risk assessment

For non-carcinogenic risks associated with PAHs in the water samples collected from the study area, average daily dosage by dermal contact (ADD_derm_) in mg/kg/day was calculated using Eq. (1).

1 $${\text{ADD}}_{\text{derm}} = \frac{{C \times {\text{SA}} \times {\text{Kp}} \times {\text{ET}} \times {\text{EF}} \times {\text{ED}} \times {\text{CF}}}}{{{\text{BW}} \times {\text{AT}}}}$$

where ADD_derm_ is the average daily doses by dermal contact (mg/kg/day); *C* represents the pollutant’s concentration in the water sample (mg/L); EF is the exposure frequency (250 days/year for dermal absorption); ED stands for the exposure duration (adult: 30 years; child: 6 years); BW is the average body weight (adult: 70 kg; child: 15 kg); AT is the average time, i.e. ED × 365 days (adult: 10,950 days; child: 2190 days); SA means the exposed skin area (adult: 18,000 cm^2^; child: 6600 cm^2^); Kp (cm/h) represents the dermal permeability coefficient (Nap: 6.90E−02; Phe: 2.70E−01; Flt: 3.60E−01; BaA: 8.10E−01; Chry: 8.10E−01; BbF: 1.20E+00; BaP: 1.20E+00; DiahA: 2.70E+00; InPy: 1.90E+00); ET means the exposure time of shower and bathing (adult: 0.58 h/day; child: 1 h/day); and CF stands for unit conversion factor (L/1000 cm). The values used in these calculations were mainly obtained from Department of Environmental Affairs (DEA), South Africa and US EPA guidelines (Gerba 2006; Kamunda et al. 2016; US EPA 1989, 2004, 2015; ATSDR 2005; DEA 2010; DTSC 2014; Feng et al. 2016; Wang et al. 2018).

The hazard quotient (HQ) was thereafter determined for the non-carcinogenic PAHs by multiplying the ADD with reference dose (RfD) for each contaminant as shown in Eq. (2) in accordance with US EPA Hazard Quotient Risk Calculation model RISC 4.02 (US EPA 1989), while hazard index (HI) was calculated as the sum of the HQs for all the congeners in each sample as shown in Eq. (3) (Wei et al. 2015).

2 $${\text{HQ}} = \frac{{\text{ADD}}}{{\text{RfD}}}$$

3 $${\text{HI}} = \sum {\text{HQs}}$$

RfD, which is the dermal reference dose for each organic pollutant and was only available for 6 of the 16 priority PAHs as shown in Table 4.

### Carcinogenic risk assessment

The carcinogenic risks in this study were determined by the estimation of LADD (mg/kg/day), incremental lifetime cancer risk (ILCR) and risk index (RI) in the water samples. Equations (1) was employed in the same manner to determine the LADD (by dermal contact), except that AT = 25,550 (i.e. 70 × 365 days) was used for lifetime assessment for the two age groups. LADD_derm_ and ILCR_derm_ were not computed for BkF because no Kp value was available for the compound. ILCR refers to the incremental probability of a person to develop cancer over a lifetime by means of exposure to a possible carcinogen. Therefore, ILCR and RI were calculated for the carcinogenic PAHs using Eqs. (4) and (5) in accordance with US EPA guidelines as shown below (Jamhari et al. 2014; US EPA 1989, 2015).

4 $${\text{ILCR}} = {\text{LADD}} \times {\text{CSF}}$$

5 $${\text{RI}} = \sum {\text{ILCR}}$$

where CSF is the cancer slope factor for individual congener. CSF for BaP is 7.3 mg/kg/day (US EPA 2015). The slope factors for other PAHs were thereafter estimated from that of BaP by multiplying the value with the respective toxic equivalent factor (TEF) for each of them as shown in Table 6 (Kumar et al. 2015; Wei et al. 2015; IARC 2006).

### Evaluation of toxic and mutagenic equivalent quotients of PAHs in the sediment samples

The toxic equivalent quotient (TEQ) or carcinogenic potential of the high molecular weight PAHs and mutagenic equivalent quotient (MEQ), otherwise referred to as the capacity of each congener to cause modification in human’s DNA (deoxyribonucleic acid), were calculated by multiplying their individual toxic equivalent factor (TEF) and mutagenic equivalent factor (MEF) with the mean concentration of each PAH in the sediment samples as shown in Eqs. (6) and (7) (Benson et al. 2017; Lerda 2011; CCME 2010).

6 $${\text{TEQ}} = \varSigma C_{n} \cdot {\text{TEF}}_{n}$$

7 $${\text{MEQ}} = \varSigma C_{n} \cdot {\text{TEF}}_{n}$$

where *C*_*n*_ = concentration of the individual PAH congener (*n*) in the mixture, and TEF_*n*_ = toxic equivalence factor for individual PAH congener (*n*). MEF_*n*_ = mutagenic equivalent factor for individual PAH congener (*n*).

### Statistical analyses

IBM SPSS version 20 software was used for the descriptive statistics of data gathered. Analysis of variance (ANOVA) and correlation were carried out to assess the variation and relationship between and within groups of data, while the statistical significance was considered for p < 0.05 (Benson et al. 2017; Jiao et al. 2011).

## Results

### Levels of polycyclic aromatic hydrocarbons (PAHs) in the water samples

The results obtained in the determination of PAHs in the water samples from Algoa Bay are presented in Table 2. All the 16 congeners of PAHs under investigation were detected at both levels (surface and bottom), although at different frequencies. Fluorene and chrysene were the most frequently found congeners at 98% and 80% in the surface and bottom waters, respectively (Table 2). The total concentration of these contaminants in the surface and bottom water samples ranged from 12.78 to 78.94 and 1.20 to 90.51 μg/L, respectively. Acenaphthylene had the highest individual PAH concentration in the surface water (9.95 ± 0.13 μg/L), while dibenzo(a,h)anthracene recorded the highest at the bottom level (10.16 ± 1.40 μg/L). Naphthalene, chrysene and benzo(a)pyrene exceeded the maximum allowable concentrations (MAC) of 1 μg/L, 0.1 μg/L and 0.01 μg/L for marine waters at both water depths (British Columbia 1993). The Agency for Toxic Substances and Disease Registry (ATSDR) in 2009 had, however, recommended MAC of 0.2 μg/L for BaP (the most toxic PAH) in the aquatic systems, which was equally surpassed at the two water levels in this study, hence signalling a serious health risk for the marine animals in the waterbody.

**Table 2:** Concentrations of the 16 priority PAHs in the water and sediment samples from Algoa Bay

PAHs	Surface water			Bottom water			Sediment			
	Range (µg/L)	Mean (µg/L)	FD (%)	Range (µg/L)	Mean (µg/L)	FD (%)	Range (µg/kg)	Mean (µg/kg)	ERL (µg/kg)	FD (%)
Naphthalene	ND–5.53	4.22 ± 0.15	40	ND–11.05	5.62 ± 0.56	28	ND–541	235 ± 26.38	160	38
Acenaphthylene	ND–10.71	9.95 ± 0.13	20	ND–14.49	7.07 ± 1.11	32	ND–73.21	50.65 ± 4.91	44	25
Acenaphthene	ND–13.10	4.34 ± 1.07	64	ND–18.94	4.41 ± 1.28	68	ND–264	59.29 ± 15.08	16	50
Fluorene	ND–7.17	3.68 ± 0.39	92	ND–12.04	4.97 ± 0.58	72	ND–1085	540 ± 77.22	19	92
Anthracene	ND–14.14	5.61 ± 0.80	56	ND–14.89	6.87 ± 0.92	64	ND–902	334 ± 58.43	85.3	67
Phenanthrene	ND–15.11	9.01 ± 1.03	20	ND–14.26	7.97 ± 0.96	32	ND–239	97.68 ± 13.22	240	67
Fluoranthene	ND–2.77	0.58 ± 0.19	32	ND–0.62	0.17 ± 0.05	20	ND–643	411 ± 57.43	600	29
Pyrene	ND–7.51	2.54 ± 0.39	44	ND–6.63	2.10 ± 0.43	56	ND–565	108 ± 26.38	665	67
Benzo(a)Anthracene	ND–4.95	1.46 ± 0.23	80	ND–22.81	3.71 ± 1.02	76	ND–1100	251 ± 63.93	261	96
Chrysene	ND–24.66	5.78 ± 1.42	80	ND–17.67	5.86 ± 0.95	80	ND–902	257 ± 47.38	384	92
Benzo(b)Fluoranthene	ND–7.81	2.19 ± 0.38	76	ND–7.91	2.64 ± 0.46	68	ND–804	187 ± 40.80	NA	92
Benzo(k)Fluoranthene	ND–14.48	3.36 ± 0.84	72	ND–11.65	4.12 ± 0.76	60	ND–1021	198 ± 59.08	NA	79
Benzo(a)Pyrene	ND–7.04	2.26 ± 0.32	60	ND–8.52	2.46 ± 0.47	68	ND–1095	199 ± 49.71	430	75
Dibenzo(a,h)Anthracene	ND–13.29	8.59 ± 0.95	28	ND–20.86	10.16 ± 1.40	32	ND–1114	308 ± 78.07	63.4	42
Indeno(1,2,3-Cd)Pyrene	ND–11.38	4.09 ± 0.77	68	ND–15.11	5.63 ± 0.77	56	ND–1099	268 ± 55.65	NA	54
Benzo(g,h,i)Perylene	ND–16.33	5.89 ± 0.90	36	ND–16.72	7.50 ± 0.99	40	ND–5235	885 ± 300	NA	46
∑PAHs	12.78–78.94	74.59 ± 9.7	–	1.20–90.51	81.28 ± 12.7	–	1168–10,469	4313 ± 964		–
∑LMW	3.33–65.76	36.35 ± 3.43	–	0.32–52.98	36.91 ± 5.4	–	161–1911	1316 ± 195	–	–
∑HMW	4.96–68.79	38.24 ± 6.26	–	0.88–72.92	44.36 ± 7.3	–	119–10,308	2996 ± 769	–	–
∑cPAHs	4.96–64.32	29.02 ± 4.85	–	0.88–70.56	34.59 ± 5.83	–	119–9743	1666 ± 395	–	–

∑*PAHs* sum of polycyclic aromatic hydrocarbons, ∑*cPAHs* sum of carcinogenic polycyclic aromatic hydrocarbons, ∑*LMW* sum of low molecular weight PAHs, ∑*HMW* sum of high molecular weight PAHs, *FD* frequency of detection, *ERL* effects range low, *ND* not detected, *NA* not applicable (Aagh et al. 2016; Adeniji et al. 2018

**Fig. 2:**
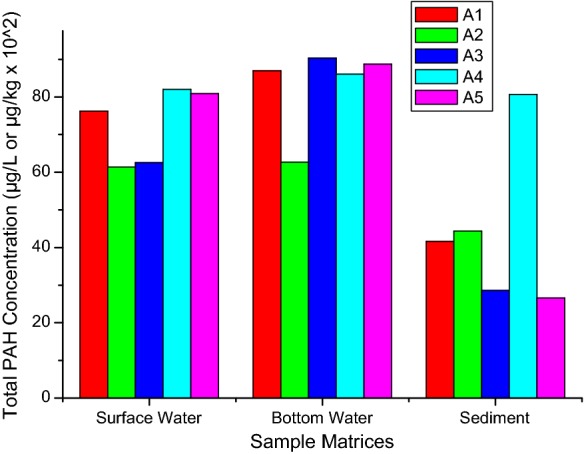
Spatial variability of PAHs in the surface and bottom water of Algoa Bay

**Fig. 3:**
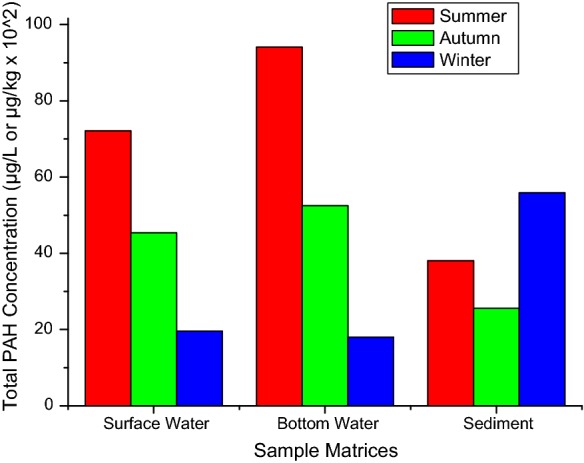
Seasonal concentrations of PAHs in the surface and bottom water samples from Algoa Bay

Generally, mean level of the 16 PAHs in the bottom water (81.28 ± 12.7 μg/L) was higher than that obtained at the surface (74.59 ± 9.7 μg/L) as was the case in the Western Harbour and El-Mex Bay along the Alexandria Coast of Egyptian Mediterranean Sea (Shreadah et al. 2013). None of the 16 analytes under investigation was found with statistically significant concentration across the 5 sampling locations (Fig. 2). The mean total concentrations of PAHs at the two water levels exceeded the permissible limits of 30 μg/L for marine waters (DoE 2003) and were generally higher in summer than other two seasons, with a noticeable trend of distribution given as follows: summer > autumn > winter (Fig. 3). This was in agreement with the reports published earlier, in which the concentrations were also lower in winter (Klages and Bornman 2003; Adeniji et al. 2017).

### Levels of PAHs in the sediment samples

The concentrations of the 16 PAHs determined in the Algoa Bay sediments are summarized in Table 2. The total concentration ranged from 1168 to 10,469 µg/kg. Fluorene (92%), benzo(a)anthracene (96%), chrysene (92%) and benzo(b)fluoranthene (92%) were more frequently detected amongst the 16 priority PAHs determined. While benzo(g,h,i)perylene was found with the highest mean concentration of 885 ± 300 µg/kg, the least mean value in the sediment was recorded by acenaphthylene (50.65 ± 4.91 µg/kg). The three dominant congeners {i.e. benzo(g,h,i)perylene, fluorene and fluoranthene} constituted 42.6% of the total PAHs in the samples analysed (Table 2). Total mean concentration of all the contaminants (4313 ± 964 µg/kg) was slightly above the effects range low (ERL) value of 4000 μg/kg recommended for the assessment of PAHs in the sediment. High molecular weight PAHs (H-PAHs) constituted up to 69.5% of the total levels obtained, corroborating many findings in the literature (Gorleku et al. 2014; Mirza et al. 2012).

The least value (2664 µg/kg) was observed at sampling station A5 and highest concentration of 8069 µg/kg at station A4, of which 35% was contributed by benzo(g,h,i)perylene (Fig. 2). The levels recorded at stations A1 and A2 were relatively the same. The organic carbon contents of the sediment samples from Algoa Bay ranged from 1.06 to 2.05% as documented earlier by Adeniji et al. (2017). The values were very consistent with previous report by Klages and Bornman (2005). The pattern of distribution across the season was similar to that observed in the water column, except that the extremely high level of benzo(g,h,i)perylene in winter raised the total concentration in that season above the values obtained in other seasons. Thus, the decreasing order of their concentrations in the sediment across the three seasons is given as follows: winter > summer > autumn (Fig. 3).

### Distribution of ring sizes and sources diagnostic ratios of PAHs in Algoa Bay

**Fig. 4:**
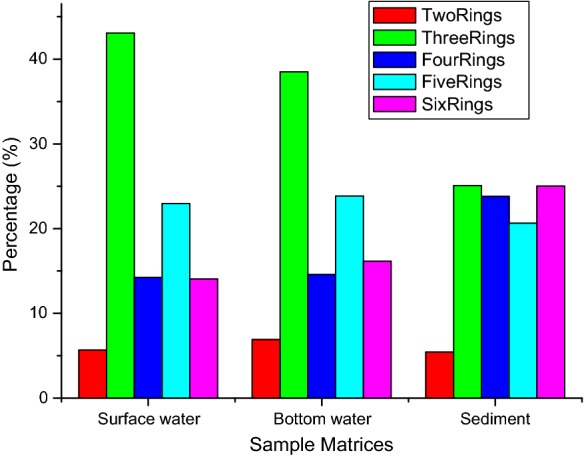
Ring sizes of PAHs in Algoa Bay

In this study, two and three rings congeners which are regarded as the low molecular weight PAHs (L-PAHs) account for 48.7%, 45.4% and 30.5% of the total PAHs in the surface water, bottom water and sediment matrices, respectively. The levels recorded in the water column were in agreement with past findings (Kafilzadeh et al. 2011; Manoli and Samara 1999; Adeniji et al. 2017). The distribution of ring sizes revealed the dominance of 3-ring PAHs in both water and sediment samples. Aside this, 4 and 5 aromatic rings were more abundant in the water column as was also reported by Hajisamoh (2013). However, the pattern of distribution in the sediment showed the prevalence of 4 and 6 aromatic rings, which account for 69.5% of the total PAHs in the sediment compartment (Fig. 4) (Jamhari et al. 2014; Hussein et al. 2016; Adeniji et al. 2017; Tobiszewski and Namiesnik 2012). This was in agreement with the reports of Kennicutt et al. (1994).

Ten molecular diagnostic ratios were used to identify possible sources of PAHs in the bay as shown in Table 3. Those ratios assisted in distinguishing between the contributions from pyrogenic (incomplete combustion of fossil fuel and vehicular exhaust emission) and petrogenic (unburnt crude oil and its other products which include kerosene, gasoline, diesel, asphalt and lubricating oil) origins of PAHs in the study area (Jamhari et al. 2014). Six of the ratios (Phen/Anth, Chry/BaA, Anth/Anth+Phe, BaA/BaA+Chry, InPy/InPy+BghiP and LMW/HMW) in all the environmental media investigated pointed to accumulation of the pollutants from pyrolytic sources, while the other four (Flt/Flt+Pyr, Flt/Pyr, An/178, BaA/228 and LMW/HMW) revealed mixed sources (Table 3) (Adeniji et al. 2018).

**Table 3:** Molecular diagnostic ratios and possible sources of PAHs in the water and sediment samples from Algoa Bay

PAHs	Petrogenic	Pyrolytic	Surface water	Bottom water	Sediment
Phen/Anth	> 15	< 10	1.88	1.67	0.49
Chry/BaA	< 0.4	> 0.9	14.29	4.66	1.95
Anth/Anth+Phen	< 0.1	> 0.1	0.83	0.75	0.80
Flt/Flt+Pyr	< 0.4	> 0.4	0.17	0.16	0.70
Flt/Pyr	< 1.0	> 1.0	0.34	0.23	4.83
Anth/178	< 0.1	≥ 0.1	0.03	0.04	1.88
BaA/228	< 0.2	0.2–0.35	0.01	0.02	1.10
BaA/BaA+Chry	< 0.2	0.33	0.37	0.37	0.51
InPy/InPy+BghiP	< 0.2	> 0.2	0.76	0.67	0.55
LMW/HMW	> 1.0	< 1.0	0.95	0.83	0.44

*Phen* phenanthrene, *Anth* anthracene, *Chry* chrysene, *BaA* benzo[a]anthracene, *Flt* fluoranthene, *Pyr* pyrene, *InPy* indeno(123,cd)pyrene, *BghiP* benzo[g,h,i]perylene, *LMW* low molecular weight, *HMW* high molecular weight (Moyo et al. 2013; Adeniji et al. 2018)

### Health risk assessment to humans

The level of risk (non-carcinogenic and carcinogenic) posed to people who are exposed to the organic micro-pollutants present in the water of Algoa Bay by dermal contact was evaluated. The HQs and ILCRs due to exposure of humans to PAHs in the bay were estimated from ADD. The results are presented in Tables 4 and 5.

**Table 4:** Hazard quotients (HQs) of PAHs in the water and sediment samples from Algoa Bay

PAHs	RfD	Dermal absorption			
		Surface water		Bottom water	
		Adults (× 10^−4^)	Children (× 10^−3^)	Adults (× 10^−4^)	Children (× 10^−4^)
Naphthalene	0.02	14.86	4.39	19.8	58.41
Fluorene	0.04	–	–	–	–
Anthracene	0.04	–	–	–	–
Phenanthrene	0.04	60.36	17.81	54.96	162.13
Fluoranthene	0.04	6.21	1.83	1.56	4.6
Benzo(g,h,i)perylene	0.04	–	–	–	–
HI		81.42	24.02	76.31	225.14

*PAHs* polycyclic aromatic hydrocarbons, *RfD* reference dose, *HI* hazard index (Wei et al. 2015)

**Table 5:** Incremental lifetime carcinogenic risk (ILCR) of cPAHs in the water and sediment samples from Algoa Bay

cPAHs	CSF	Dermal absorption			
		Surface water		Bottom water	
		Adults (× 10^−5^)	Children (× 10^−6^)	Adults (× 10^−5^)	Children (× 10^−6^)
Benzo(a)anthracene	0.73	3.95	23.3	9.61	56.71
Chrysene	0.073	1.55	9.13	1.52	8.95
Benzo(b)fluoranthene	0.73	8.84	52.15	10.14	59.79
Benzo(k)fluoranthene	0.73	–	–	–	–
Benzo(a)pyrene	7.3	89.96	530.77	94.3	556.42
Dibenzo(a,h)anthracene	7.3	77	454.33	87.68	517.32
Indeno(1,2,3-cd)pyrene	0.73	26.65	157.22	34.19	201.73
RI		207.93	1226.89	237.43	1400.92

*PAHs* polycyclic aromatic hydrocarbons, *CSF* cancer slope factor, *cPAHs* carcinogenic PAHs, *RI* risk index (Wang et al. 2009; CCME 2010; Kumar et al. 2015; Wei et al. 2015; Benson et al. 2017)

HQ or HI < 1 indicates no significant non-carcinogenic risk, while the effect will be considered substantial when the value exceeds 1 (i.e. HQ or HI > 1) (Wei et al. 2015; US EPA 2001; Titilawo et al. 2018; Siyue and Quanfa 2010). The HQs obtained in this study in relation to exposure of people to the contaminants in the surface and bottom water via skin absorption were all lesser than 1 as shown in Table 4, suggesting there would be no likelihood of any non-carcinogenic effects as a result of dermal contact. HI, which is the sum of HQs in each category generally ranged between 0.008 and 0.024, with an average of 0.016 which was also below the United States Environmental Pollution Agency’s recommended limit (Benson et al. 2017; ATSDR 2013).

ILCR of one in a million population (1 × 10^−6^) is considered acceptable or insignificant by US EPA. Values above 1 × 10^−5^ but lesser than 1 × 10^−4^ may although be tolerable but not recommendable, while values up to 1 × 10^−4^ or higher portend serious cancer risk in humans (Benson et al. 2017; AEG 2015; Man et al. 2013). ILCRs by dermal exposure in Algoa Bay, especially through washing, showering and swimming, being a recreational environment were evaluated (Karyab et al. 2016). The dermal ILCRs in the water phase were generally in the range of 8.95 × 10^−6^–9.43 × 10^−4^. Result estimates showed that carcinogenic RIs, being sum of ILCRs by skin contact for children in the surface and bottom water, were 0.0012 and 0.0014, respectively (Table 5). In the same vein, the RIs recorded for adults by dermal absorption in the two matrices were 0.0021 and 0.0024, correspondingly.

### Carcinogenic (TEQ) and mutagenic (MEQ) equivalents of PAHs in the sediment samples

The estimated carcinogenic and mutagenic potentials of the seven congeners of PAHs usually regarded as probable human carcinogens and BghiP are presented in Table 6. The carcinogenic PAHs (cPAHs) have been reportedly identified as marker compounds for vehicular emissions (Jamhari et al. 2014; Oanh et al. 2000). In this study, they contributed a percentage of 69.5% of the total concentration of PAHs determined in the sediments. The toxic equivalent quotient (TEQ) calculated for the 8 PAHs (including BghiP) revealed that BaP and DiahA, the two most toxic PAHs, were exceptionally higher in the sediments, in agreement with the report of Kumar et al. (2014). The duo contributed roughly 83% of the total TEQ in the samples analysed. Similarly, the mutagenic equivalent quotient (MEQ) for the compounds was generally low, except for DiahA that was found extremely higher, recording up to 13% of the overall MEQ obtained (Table 6), while the contributions from other compounds to both TEQ and MEQ were very negligible.

**Table 6:** TEQ and MEQ of PAHs in the sediment samples from Algoa Bay

PAHs	TEF	Calculated TEQ (µg/kg)	MEF	Calculated MEQ (µg/kg)
Benzo(a)anthracene	0.1	26	0.082	22
Chrysene	0.01	3	0.017	5
Benzo(b)fluoranthene	0.1	19	0.25	48
Benzo(k)fluoranthene	0.1	19	0.11	21
Benzo(a)pyrene	1	194	1.0	194
Dibenzo(a,h)anthracene	1	313	0.29	91
Indeno(1,2,3-cd)pyrene	0.1	29	0.31	88
Benzo(g,h,i)perylene	0.01	9	0.19	168
Total		611		636

*PAHs* polycyclic aromatic hydrocarbons, *TEF* toxic equivalent factor, *TEQ* toxic equivalent quotient, *MEF* mutagenic equivalent factor, *MEQ* mutagenic equivalent quotient (WDNR 2003; Wei et al. 2015; Benson et al. 2017)

## Discussion

### Levels of polycyclic aromatic hydrocarbons (PAHs) in the water samples

The observed higher concentrations of PAHs in the bottom water could be related to the accumulation of high molecular PAHs in the sediment as a result of their decreasing solubility in the water phase (Dhananjayan et al. 2012). The pollutants in the water column are most likely from combustion engines of ships and vehicles, various industrial origins, inveterate oil contamination at the study sites, and/or urban run-off. High level of PAHs in the marine water is usually dangerous, as it could smother the benthic organisms and as well bring about cellular poisoning in them (Klages and Bornman 2003). The high level determined in summer could be linked with influx of run-off after heavy rainfall from Port Elizabeth, being a metropolitan and industrial city, as well as other neighbouring towns (Yahaya et al. 2017; Kumar et al. 2014).

The PAHs levels in the water column of Algoa Bay were compared with values reported in other studies across the globe. The results were very similar to the levels found in Tema Harbour, Ghana (Gorleku et al. 2014), Daya Bay, China (Zhou and Maskaoui 2003), coastal belt of Ghana (Essumang 2010) and Persian Gulf coastal areas (Sinaei and Maschinchian 2014). However, they were several folds higher than those reported for samples collected from Todos Os Santos Bay, Brazil (Celino et al. 2012), Mumbai Harbour Line, India (Dhananjayan et al. 2012) and Deep Bay, South China (Qiu et al. 2009).

### Levels of PAHs in the sediment samples

High percentage of H-PAHs in sediment is usually linked with sources such as combustion of fossil materials, air deposition and run-off (Doan et al. 2005). Levels of PAHs above ERL are a pointer to likely biological risk on aquatic organisms (DoE 2003; Kim et al. 1999; MacDonald et al. 2000; Adeniji et al. 2018). Of the 16 compounds determined, six (Nap, Ace, Acy, Flu, Ant and DiahA) were conspicuously higher than their ERL values (Table 2). The most worrisome of them is DiahA, which has similar toxicity as BaP (Kumar et al. 2014). However, none of the 6 PAHs exceeded the effect range median (ERM) values, indicating that the level of toxicity posed by the contaminants to the marine animals such as birds, fish, and mammals could be mild (Adeniji et al. 2018; Edokpayi et al. 2016).

Undoubtedly, the activities at the Port Elizabeth and Ngqura Harbours might be contributing immensely to the amount recorded at the sampling points 1 and 2, respectively (AES 1999; CSIR 2007). Other possible factors may include the urban run-off, industrial effluents pollution threats from Port Elizabeth metropolis and the Coega Industrial Development Zone, as well as sewage outfall from Uitenhage/Despatch Sewage Treatment Works entering the bay through its estuaries (Klages et al. 2011; AES 1999). Oils from ship transportation could as well impact the environment negatively to some extent to the detriment of the community of African penguins especially and other marine animals in the area (Klages et al. 2011; SST 2006; CSIR 2007; Lee 2016). However, it is unclear what could have raised the concentration of BghiP at Alexandria Dune Fields to such a high level, although diffusion of pollutants from other nearby stations due to intense mixing of water occasioned by strong currents in the area is very possible (Klages and Bornman 2003).

The concentrations of these organic micro-pollutants were expectedly higher in the sediments than the water column, as hypothesized by Klages and Bornman (2003), being a potential reservoir for organic compounds that are resistant to biodegradation in the aquatic environment (Irwin et al. 1997). BghiP, which recorded the highest concentrations amongst the 16 compounds determined (especially at A4) in winter, is known to be a reliable indicator of incomplete combustion (usually from vehicle exhausts, coal fires and domestic wood) of PAHs in the aquatic environment (Stogiannidis and Laane 2015). Other possible origin of BghiP includes emission from industrial effluents, municipal waste water treatment facilities, waste incinerators and aluminium smelting. It is most often strongly attached to sediment and other solid matter in the aquatic environment (http://apps.sepa.org.uk/spripa/pages/substanceinformation.aspx?pid=236).

PAHs concentrations in this study were comparatively found within the ranges of previous reports on sediments in Cienfuegos Bay, Cuba (Tolosa et al. 2009) and Izmit Bay, Turkey (Tolun et al. 2006), but much lower than the levels obtained in Santander Bay, Northern Spain (Viguri et al. 2002), Mediterranean sea, France (Mille et al. 2007) and Mumbai Harbour Line, India (Dhananjayan et al. 2012). However, they were found higher than those reported for coastal sediments in some other regions including Masan Bay, Korea (Yim et al. 2005), Coastal of Bushehr, Persian Gulf (Mirza et al. 2011), Khure-Musa estuarine, Persian Gulf (Mirza et al. 2012) and Western Xiamen Bay, China (Zhou et al. 2000).

### Distribution of ring sizes and sources diagnostic ratios of PAHs in Algoa Bay

L-PAHs are found more in the large atmospheric particles which deposit faster (Fang et al. 2004) and are subsequently washed into the nearby waterbody by rain in the form of run-off (Nekhavhambe et al. 2014). H-PAHs (4–6 rings), however, are commonly from sources related to high-temperature processes (Abdel-Shafy and Mansour 2016; Zhang et al. 2008). They are usually observed in higher concentrations in the sediment, especially those from marine environment than in water samples, because of their resistance to degradation and increased adsorption to suspended particles (Mirza et al. 2012; Mostafa et al. 2009; Culotta et al. 2006).

Overall assessment showed that PAHs in all the environmental compartments of Algoa Bay were though from mixed sources (Gorleku et al. 2014; Dhananjayan et al. 2012), but more predominantly from pyrogenic than petrogenic origins. In accordance to Adeniji et al. (2017), pollution in Algoa Bay is much more from other anthropogenic sources than petroleum origin. This can be attributed to fossil fuel combustion from ship transportation, vehicular and industrial emission carried into the bay as run-off, domestic and industrial waste discharges, sewage outfalls, oil spills from large industrial ships, pumping of bulges into water from ship, leakages arising from the transfer from one ship to another or from the two-stroke engines and release of black smokes by ships while passing (Jamhari et al. 2014; Klages et al. 2011; SST 2006; Gorleku et al. 2014; Mirza et al. 2012; Adeniji et al. 2018; Dhananjayan et al. 2012; NMBT 2016; Sany et al. 2014).

### Health risk assessment to humans

The HQs and HIs estimated for children and adults in the water column of the bay did not suggest any non-carcinogenic effects which are primarily noticed in the renal, pulmonary, gastrointestinal, and dermatologic systems of the human body (Benson et al. 2017; ATSDR 2013). However, the calculated carcinogenic risk indices by dermal contact in this study were generally above 10^−4^, pointing to serious cancerous effects to both adults and children. The carcinogenic effects of PAHs have been reported to be more prominent in certain parts of the body which include the gastro-intestine, liver, lung, bladder and skin (Benson et al. 2017; ATSDR 2013). It is worthy of note that adults with highest risks in this evaluation will generally be more vulnerable than children (Karyab et al. 2016). Contaminants with higher risks are BaP and DiahA (Wei et al. 2015).

### Carcinogenic (TEQ) and mutagenic (MEQ) equivalents of PAHs in the sediment samples

The TEQs obtained for BaP and DiahA in this study signified a possible cancer risk to those who may be exposed to the marine sediment (Benson et al. 2017; Hussein et al. 2016; Van den Berg et al. 2006; WDNR 2003; Salem et al. 2014), and the MEQ for DiahA showed that the congener could trigger a number of other health issues excluding cancer to an extent in humans after exposure. Such problems may include low intelligent quotient, lung diseases, birth defects, impotence and many others (Benson et al. 2017; Hsu et al. 2014). Since other congeners did not record any significant contribution, hence the overall potential risk due to cPAHs in Algoa Bay sediment is considered to be minimal (Błaszczyk et al. 2017).

## Conclusion

The results obtained in this study showed that the bay is fairly contaminated with polycyclic aromatic hydrocarbons which are predominantly from pyrogenic origin in all the seasons of study as indicated by the diagnostic ratios determined. The total concentrations of PAHs in the water columns were higher in summer than in other seasons in agreement with previous studies, showing a trend of distribution as thus presented: summer > autumn > winter. This could be related to possible increase in maritime and road traffic in the season. The distribution order in the sediment was also similar, only that benzo(g,h,i)perylene was extremely high in winter and so raised the total level of the contaminants in that season than in summer, giving rise to an order as follows: winter > summer > autumn. Meanwhile, statistical analysis did not show any significant spatial or seasonal variation across the 5 locations in all the environmental media of study. Moreover, the health risk assessment did not reveal any non-carcinogenic risk tendency in all the matrices. However, with US EPA Hazard Quotient Risk Calculation model RISC 4.02, carcinogenic risk by dermal absorption was higher in the water medium, especially for benzo(a)pyrene and dibenzo(a,h)anthracene. It was very obvious that adults would be more vulnerable to the cancer risk than the children. In the same vein, TEQs for benzo(a)pyrene and dibenzo(a,h)anthracene (the two most toxic) were higher than expected, as well as the MEQ for dibenzo(a,h)anthracene. Therefore, it is recommended that all the activities contributing to the increased pollution in the area should be brought under control, so as to keep the environment safe for aquatic organisms and human lives that benefit from them along the food chain.

## Electronic supplementary material

Below is the link to the electronic supplementary material.

Supplementary material 1 (DOCX 87 kb)

## References

[CR1] Aagh H, Rahmanpour S, Abedi E, Arebi I, Mahdinia A (2016). Contamination of polycyclic aromatic hydrocarbons in seawater and sediments of West-Northern coasts of the Persian Gulf. Indian Journal of Geo-Marine Sciences.

[CR2] Abdel-Shafy HI, Mansour MSM (2016). A review on polycyclic aromatic hydrocarbons: Source, environmental impact, effect on human health and remediation. Egyptian Journal of Petroleum.

[CR3] Adeniji AO, Okoh OO, Okoh AI (2017). Petroleum hydrocarbon profiles of water and sediment of Algoa Bay, Eastern Cape, South Africa. International Journal of Environmental Research and Public Health.

[CR4] Adeniji, A. O., Okoh. O. O., & Okoh, A. I. (2018) Analytical methods for polycyclic aromatic hydrocarbons and their global trend of distribution in water and sediment: a review. In: Manar El-Sayed Abdul-Raouf (Eds.), Recent insights in petroleum science and engineering. ISBN 978-953-51-5321-4. 10.5772/intechopen.71163.

[CR5] AEG (Alliance Environmental Group). (2015). Method 3 Risk Characterization. File No: 5193-01-01, 2015; p. 78. Accessed 20 Dec 2016, https://www.salem.com/sites/salemma/files/uploads/oto_method_3_risk_char._10-19-15.pdf.

[CR6] AES (African Environmental Solutions). (1999) Algoa Bay Management Plan. Prepared by CLABBS Consortium, 1999; pp. 1–63. Accessed 22 Dec 2016, http://fred.csir.co.za/project/CIP_EIA/pages/Algoa_Bay_Management_Plan_Dec99.pdf.

[CR7] ATSDR (Agency for Toxic Substances and Disease Registry). (2009). Case studies in environmental medicine: toxicity of polycyclic aromatic hydrocarbons (PAHs), pp. 23–26.

[CR8] ATSDR (Agency for Toxic Substances and Disease Registry). (2012). Public Health Assessment Guidance Manual (2005 Update). Appendix G: Calculating Exposure Doses, 2012. Accessed 19 Apr 2018, https://www.atsdr.cdc.gov/hac/phamanual/appg.html.

[CR9] ATSDR (Agency for Toxic Substances and Disease Registry). (2013). Polycyclic Aromatic Hydrocarbons (PAHs): What Health Effects Are Associated with PAH Exposure? 2013. Retrieved November 29, 2016 from https://www.atsdr.cdc.gov/csem/csem.asp?csem=13&po=11.

[CR10] Benson NU, Anake AE, Adedapo WU, Fred-Ahmadu OH, Eke KP (2017). Polycyclic aromatic hydrocarbons in imported Sardinops sagax: Levels and health risk assessments through dietary exposure in Nigeria. Journal of Food Composition and Analysis.

[CR11] BirdLife International. (2009). Important bird area factsheet: Algoa Bay island nature reserve, South Africa, 2009. Accessed 12 Dec 2016, Downloaded from the Data Zone at http://www.birdlife.org.

[CR12] Błaszczyk E, Rogula-Kozłowska W, Klejnowski K, Fulara I, Mielżyńska-Švach D (2017). Polycyclic aromatic hydrocarbons bound to outdoor and indoor airborne particles (PM2.5) and their mutagenicity and carcinogenicity in Silesian kindergartens, Poland. Air Quality, Atmosphere and Health.

[CR13] Bornman, T. G. (2003.) Report on seawater quality in the port of Port Elizabeth (March). Prepared for National Ports Authority. Institute for Environmental and Coastal Management Report, *C82*, 26

[CR71] Bottomley, E. (2016) Nelson Mandela Bay’s rich marine biodiversity, 2014. Accessed 7 Dec 2016, http://blog.nmbt.co.za/blog/entry/nelson-mandela-bays-rich-marine-biodiversity.

[CR14] Brazkova M and Krastanov A. Polycyclic aromatic hydrocarbons: sources, effects and biodegradation. Hayчни Tpyдoвe Ha Pyceнcкия Унивepcитeт, 2013; Toм 52, Cepия *10*(2), 1–5.

[CR15] British Columbia. (1993). Water Quality. Ambient Water Quality Criteria for Polycyclic Aromatic Hydrocarbons (PAHs): Overview Report. Ministry of Environment, Lands and Parks, pp. 9.

[CR16] Cai S, Syage JA, Hanold KA, Balogh MP (2009). Ultra-performance liquid chromatography atmospheric pressure photo ionization-tandem mass spectrometry for high-sensitivity and high-throughput analysis of U.S. environmental protection agency 16 priority pollutants polynuclear aromatic hydrocarbons. Analytical Chemistry.

[CR17] CCME (Canadian Council of Ministers of the Environment). (2010). Canadian Soil Quality Guidelines for Carcinogenic and Other Polycyclic Aromatic Hydrocarbons (PAHs) (Environmental and Human Health Effects). Scientific Criteria Document (Revised), Publication No. 1445 [Internet]. 2010. Accessed 13 June 2016, http://www.ccme.ca/files/Resources/supporting_scientific_documents/pah_soqg_scd_1445.pdf.

[CR18] Celino JJ, Corseuil HX, Fernandes M, Hadlich GM (2012). Persistent toxic substances in surface water of Todos Os Santos Bay, Brazil. Resources and Environment.

[CR19] Coelho E, Ferreira C, Almeid CMM (2008). Analysis of polynuclear aromatic hydrocarbons by SPME-GC-FID in environmental and tap waters. Journal of the Brazilian Chemical Society.

[CR20] Cortes JE, Suspes A, Roa S, González C, Castro HE (2012). Total petroleum hydrocarbons by gas chromatography in Colombian waters and soils. American Journal of Environmental Sciences.

[CR21] CSIR (Council for Scientific and Industrial Research). (2007). Proposed extension to the container berth and construction of an administration craft basin at the Port of Ngqura. Chapter 6: Marine ecology, sediment toxicology and dredging. Draft Scoping Report, pp. 08–18.

[CR22] Culotta L, De Stefano C, Gianguzza A, Mannino MK, Orecchio S (2006). The PAH composition of surface sediments from stagnone coastal lagoon, Marsala (Italy). Marine Chemistry.

[CR23] DEA (Department of Environmental Affairs, South Africa). (2010). Framework for the Management of Contaminated Land, pp. 326.

[CR24] Dhananjayan V, Muralidharan S, Peter VR (2012). Occurrence and distribution of polycyclic aromatic hydrocarbons in water and sediment collected along the Harbour Line, Mumbai, India. International Journal of Oceanography.

[CR25] Doan, M. (2005). Concentrations of Polycyclic Aromatic Hydrocarbons in Surficial Sediments of the Fore River and Portland Harbor, Maine. A Report to the Natural Resource Damage Trustees’ Agreement Number: 604195, 2005 [Internet]. Available from: http://www.cascobayestuary.org/wp-content/uploads/2014/07/2005_focb_fore_river_pahs_report6.pdf. Accessed 15 Nov 2016.

[CR26] DoE (Department of Environment). (2003). Assessment levels for soil, sediment and water. Contaminated sites management series, Western Australia. Version 3, 21.

[CR27] DTSC (California Department of Toxic Substances Control). (2014). Human Health Risk Assessment (HHRA) Note. Hero HHRA Note Number 1, pp. 4.

[CR28] Edokpayi JN, Odiyo JO, Popoola OE, Msagati TAM (2016). Determination and distribution of polycyclic aromatic hydrocarbons in rivers, sediments and wastewater effluents in Vhembe District, South Africa. International Journal of Environmental Research and Public Health.

[CR29] ESS Laboratory. (2008). Total petroleum hydrocarbons (SW 846 Method 8100 modified). SOP No: 60_8100-mod. Revision 4, 23.

[CR30] Essumang DK (2010). Distribution, levels, and risk assessment of polycyclic aromatic hydrocarbons (PAHs) in some water bodies along the coastal belt of Ghana. The Scientific World Journal.

[CR31] Fang GC, Chang CN, Wu YS, Fu PP, Yang IL, Chen MH (2004). Characterization, identification of ambient air and road dust polycyclic aromatic hydrocarbons in Central Taiwan. Science of the Total Environment.

[CR32] Fang GC, Huang JH, Huang YL (2010). Polycyclic aromatic hydrocarbons in the Asian atmosphere during 2001e2009. Environmental Forensics.

[CR33] Feng J, Hu P, Li X, Liu S, Sun J (2016). Ecological and health risk assessment of polycyclic aromatic hydrocarbons (PAHs) in surface water from middle and lower reaches of the yellow river. Polycyclic Aromatic Compounds.

[CR34] Fourie, V. (2013). Research on white sharks in Algoa Bay pays off. SABC News Feeds, 2013. http://www.sabc.co.za/news/a/26309f0041fb971fb7b7bf1c2eddf908/Research-on-white-sharks-in-Algoa-Bay-pays-off. Published: Wednesday 27 November 2013 21:10

[CR35] Gerba CP, Pepper IL, Gerba CP, Brusseau ML (2006). Chapter 14: Risk assessment. Environmental and pollution science.

[CR36] Gorleku MA, Carboo D, Palm LMN, Quasie WJ, Armah AK (2014). Polycyclic aromatic hydrocarbons (PAHs) pollution in marine waters and sediments at the Tema Harbour, Ghana. Academia Journal of Environmental Sciences.

[CR37] Hajisamoh A (2013). Pollution levels of 16 priority PAHs in the major rivers of Southern Thailand. Research and Reviews. Journal of Chemistry.

[CR38] Hsu H, Lin M, Chen Y, Chen W, Yoon C, Chen M, Tsai P (2014). An integrated approach to assess exposure and health-risk from polycyclic aromatic hydrocarbons (PAHs) in a fastener manufacturing industry. International Journal of Environmental Research and Public Health.

[CR39] Huggett RJ, Van Veld PA, Smith CL, Hargis WJ, Vogelbein WJ, Weeks BA, Burton GA (1992). The effects of contaminated sediments in the Elizabeth River. Sediment toxicity assessment.

[CR40] Hussein RA, Al-Ghanim KA, Abd-El-Atty MM, Mohamed LA (2016). Contamination of Red Sea Shrimp (*Palaemon serratus*) with polycyclic aromatic hydrocarbons: a health risk assessment study. Polish Journal of Environmental Studies.

[CR41] IARC (International Agency for Research on Cancer). (2006). Polycyclic aromatic hydrocarbons. IARC Monogaraph 92. Lyone France, 2006. Accessed 2 Feb 2014, http://monographs.iarc.fr/ENG/Meetings/92-pahs.pdf.

[CR42] Irwin RJ, van Mouwerik M, Stevens L, Seese MD, Basham W (1997). Environmental contaminants encyclopedia.

[CR43] Jamhari AA, Sahani M, Latif MT, Chan KM, Tan HS, Khan MF, Tahir NM (2014). Concentration and source identification of polycyclic aromatic hydrocarbons (PAHs) in PM10 of urban, industrial and semi-urban areas in Malaysia. Atmospheric Environment.

[CR44] Jiao L, Zheng GJ, Minh TB, Richardson B, Chen L, Zhang Y, Yeung LW, Lam JCW, Yang X, Lam PKS, Wong MH (2009). Persistent toxic substances in remote lake and coastal sediments from Svalbard, Norwegian Arctic: levels, sources and fluxes. Environmental Pollution.

[CR45] Jiao, W., Wang, T., Khim, J. S., Luo, W., Hu, W., Naile, J. E., Giesy, J. P., & Lu Y. (2011). PAHs in surface sediments from coastal and estuarine areas of the northern Bohai and Yellow Seas, China. *Environmental Geochemistry and Health*, 10653-011-9445-8, 1–12. 10.1007/s10653-011-9445-8 22203243

[CR46] Kafilzadeh F, Shiva AH, Malekpour R (2011). Determination of Polycyclic Aromatic Hydrocarbons (PAHs) in Water and Sediments of the Kor River, Iran. Middle-East Journal of Scientific Research.

[CR47] Kamunda C, Mathuthu M, Madhuku M (2016). Health risk assessment of heavy metals in soils from Witwatersrand Gold Mining Basin, South Africa. International Journal of Environmental Research and Public Health.

[CR48] Karyab H, Yunesian M, Nasseri S, Rastkari N, Mahvi A, Nabizadeh R (2016). Carcinogen risk assessment of polycyclic aromatic hydrocarbons in drinking water, using probabilistic approaches. Iranian Journal of Public Health.

[CR49] KDHE (Kansas Department of Health and Environment). (2015). Kansas method for the determination of mid-range hydrocarbons (MRH) and high-range hydrocarbons (HRH). Revision 2015; 1.0, 18–20.

[CR50] Kennicutt MC, Wade TL, Presley BJ, Requejo AG, Brooks JM, Denoux GJ (1994). Sediment contaminants in Casco Bay, Maine: inventories, sources, and potential for biological impact. Environmental Science and Technology.

[CR51] Kennish MJ (1994). Pollution in estuaries and coastal marine waters. Journal of Coastal Research.

[CR52] Kim GB, Maruya KA, Lee RF, Lee JH, Koh CH, Tanabe S (1999). Distribution and sources of polycyclic aromatic hydrocarbons in sediments from Kyeonggi Bay, Korea. Marine Pollution Bulletin.

[CR53] Klages N, Jegels J, Schovell I, Vosloo M (2011). Nelson Mandela Bay Municipality State of Environment Report.

[CR54] Klages NTW, Bornman TG (2003). Port of Ngqura marine biomonitoring programme. Annual Report 2002–2003. Institute for Environmental & Coastal Management.

[CR55] Klages NTW, Bornman TG (2005). Port of Ngqura marine biomonitoring programme. Winter 2005. Institute for Environmental & Coastal Management.

[CR56] Kumar V, Kothiyal NC, Saruchi (2015). Analysis of polycyclic aromatic hydrocarbon toxic equivalency factor and related carcinogenic potencies in roadside soil within a developing city of Northern India. Polycyclic Aromatic Compounds.

[CR57] Kumar V, Kothiyal NC, Saruchi Mehra R, Parkash A, Sinha RR, Tayagi SK, Gaba R (2014). Determination of some carcinogenic PAHs with toxic equivalency factor along roadside soil within a fast developing northern city of India. Journal of Earth System Science.

[CR58] Lee, J. (2016) Endangered African Penguins under threat following oil spill. SABC News Feeds, 2016. http://www.sabc.co.za/news/a/b540c1004df610f685d3f546a0a81a58/Endangered-African-Penguins-under-threat-following-oil-spill-20160822 Published: Monday 22 August 2016 19:38.

[CR59] Lerda, D. (2011). Polycyclic aromatic hydrocarbons (PAHs) Factsheet, 4th Edn. JRC Technical Notes, 2011; 66955-2011, pp. 6–13.

[CR60] Ma J, Xiao R, Li J, Yu J, Zhang Y, Chen L (2010). Determination of 16 polycyclic aromatic hydrocarbons in environmental water samples by solid-phase extraction using multi walled carbon nanotubes as adsorbent coupled with gas chromatography-mass spectrometry. Journal of Chromatography A.

[CR61] MacDonald DD, Ingersoll CG, Berger TA (2000). Development and evaluation of consensus-based sediment quality guidelines for freshwater ecosystems. Archives of Environmental Contamination and Toxicology.

[CR62] Man YB, Kang Y, Wang HS, Lau W, Li H, Sun XL, Giesy JP, Chow KL, Wong MH (2013). Cancer risk assessments of Hong Kong soils contaminated by polycyclic aromatic hydrocarbons. Journal of Hazardous Materials.

[CR63] Manoli E, Samara C (1999). Polycyclic aromatic hydrocarbons in natural waters: Sources, occurrence and analysis. Trends in Anaytical Chemistry.

[CR64] Mille G, Asia L, Guiliano M, Malleret L, Doumenq P (2007). Hydrocarbons in coastal sediments from the Mediterranean Sea (Gulf of Fos area, France). Marine Pollution Bulletin.

[CR65] Mirza R, Faghiri I, Abedi E (2012). Contamination of polycyclic aromatic hydrocarbons in surface sediments of Khure-Musa Estuarine, Persian Gulf. World Journal of Fish and Marine Sciences.

[CR66] Mirza R, Mohammady M, Dadoloahi A, Safahieh AR, Savari A, Hajeb P (2011). Polycyclic aromatic hydrocarbons in seawater, sediment and oyster (*Saccostrea cucullata*) from the Northern Part of the Persian Gulf (Bushehr Province). Water, Air, and Soil pollution.

[CR67] Mostafa AR, Terry LW, Stephen TS, Barakat AO (2009). Distribution and characteristics of polycyclic aromatic hydrocarbons (PAHs) in sediments of Hadhramout coastal area, Gulf of Aden. Marine Systems.

[CR68] Motsara MR, Roy RN (2008). Guide to laboratory establishment for plant nutrient analysis. FAO Fertilizer and Plant Nutrition Bulletin.

[CR69] Moyo S, McCrindle R, Mokgalaka N, Myburgh J, Mujuru M (2013). Source apportionment of polycyclic aromatic hydrocarbons in sediments from polluted rivers. Pure and Applied Chemistry.

[CR70] Nekhavhambe TJ, van Ree T, Fatoki OS (2014). Determination and distribution of polycyclic aromatic hydrocarbons in rivers, surface runoff, and sediments in and around Thohoyandou, Limpopo Province. South Africa. Water SA.

[CR72] NMBT (Nelson Mandela Bay Tourism). Algoa Bay Hope Spot. 2016. Accessed 10 Dec 2016, http://www.nmbt.co.za/algoa_bay_hope_spot.html.

[CR73] Oanh NTK, Reutergardh LB, Dung NT, Yu MH, Yao WX, Co HX (2000). Polycyclic aromatic hydrocarbons in the airborne particulate matter at a location 40KM north of Bangkok, Thailand. Atmospheric Environment.

[CR74] Ogunfowokan AO, Asubiojo OI, Fatoki OS (2003). Isolation and determination of polycyclic aromatic hydrocarbons in surface runoffs and sediments. Water, Air, and Soil pollution.

[CR75] Olatunji OS, Fatoki OS, Opeolu BO, Ximba BJ (2014). Determination of polycyclic aromatic hydrocarbons [PAHs] in processed meat products using gas chromatography—Flame ionization detector. Food Chemistry.

[CR76] Olutona GO, Oyekunle JAO, Ogunfowokan AO, Fatoki OS (2016). Assessment of polybrominated diphenyl ethers in sediment of Asunle stream of the Obafemi Awolowo University, Ile-Ife, Nigeria. Environmental Science and Pollution Research.

[CR77] Omores RA, Wewers F, Ikhide PO, Farrar T, Giwa A (2017). Spatio–temporal distribution of polycyclic aromatic hydrocarbons in Urban Soils in Cape Town, South Africa. International Journal of Environmental Research.

[CR78] Pavlova A, Ivanova R (2003). Determination of petroleum hydrocarbons and polycyclic aromatic hydrocarbons in sludge from wastewater treatment basins. Journal of Environmental Monitoring.

[CR79] Prabhukumar G, Pagilla K (2010). Polycyclic aromatic hydrocarbons in urban runoff—sources, sinks and treatment: a review.

[CR80] Qiu YW, Zhang G, Liu GQ, Guo LL, Li XD, Wai O (2009). Polycyclic aromatic hydrocarbons (PAHs) in the water column and sediment core of Deep Bay, South China. Estuarine, Coastal and Shelf Science.

[CR81] Sakuma T, Leigh D, Seto C, Schreiber A, Wittrig R (2011). Analysis of polycyclic aromatic hydrocarbons (PAH), alkylated derivatives, and photo-degradation products in environmental and food samples using LC-FLD-MS/MS with Q TRAP^®^ Technology. Food and environmental.

[CR82] Salaudeen T, Okoh O, Agunbiade F, Okoh A (2018). Fate and impact of phthalates in activated sludge treated municipal wastewater on the water bodies in the Eastern Cape, South Africa. Chemosphere.

[CR83] Salem D, Morsy F, El-Nemr A, El-Sikaily A, Khaled A (2014). The monitoring and risk assessment of aliphatic and aromatic hydrocarbons in sediments of the Red Sea, Egypt. The Egyptian Journal of Aquatic Research.

[CR84] Sany, S. B. T., Rezayi, M., Hashim, R., Salleh, A., Mehdinia, A., & Safari, O. (2014) Polycyclic aromatic hydrocarbons in coastal sediment of Klang Strait, Malaysia: Distribution pattern, risk assessment and sources. *PLoS ONE*, *e94907*; *9*(4), 1–14. www.plosone.org. 10.1371/journal.pone.0094907 PMC3991632 24747349

[CR85] Schwarz G, BaÈumler S, Block A, Felsenstein FG, Wenzel G (2004). Determination of detection and quantification limits for SNP allele frequency estimation in DNA pools using real time PCR. Nucleic Acids Research.

[CR86] Shreadah MA, Abdel Moneim MI, Said TO, Fathallah EMI, Mahmoud ME (2013). PAHs in seawater of the semi-closed areas along the Alexandria Coast of Egyptian Mediterranean Sea. Journal of Environmental Protection.

[CR87] Sinaei, M., & Mashinchian, A. (2014). Polycyclic aromatic hydrocarbons in the coastal sea water, the surface sediment and Mudskipper Boleophthalmus dussumieri from coastal areas of the Persian Gulf: source investigation, composition pattern and spatial distribution. *Journal of Environmental Health Science and Engineering, 12*, 59 http://www.ijehse.com/content/12/1/59. 10.1186/2052-336X-12-59 PMC3995868 24612928

[CR88] Siyue L, Quanfa Z (2010). Risk assessment and seasonal variations of dissolved trace elements and heavy metals in the Upper Han River, China. Journal of Hazard Material.

[CR89] SST (Sustainable Seas Trust). Algoa Bay conservation, 2006. http://www.sst.org.za/hope-spots/algoa-bay-hope-spot–2/algoa-bay-hope-spot-details. Accessed 6 Dec 2016.

[CR90] Stogiannidis E, Laane R (2015). Source characterization of polycyclic aromatic hydrocarbons by using their molecular indices: An overview of possibilities. Reviews of Environmental Contamination and Toxicology.

[CR91] Titilawo Y, Adeniji A, Adeniyi M, Okoh A (2018). Determination of levels of some metal contaminants in the freshwater environments of Osun State, Southwest Nigeria: A risk assessment approach to predict health threat. Chemosphere.

[CR92] Tobiszewski M, Namiesnik J (2012). PAHs diagnostic ratios for the identification of pollution emission sources. Environmental Pollution.

[CR93] Tolosa I, Albernas MM, Hernandez ACM (2009). Inputs and sources of hydrocarbons in sediments from Cienfuegos bay. Cuba. Marine Pollution Bulletin.

[CR94] Tolun L, Martens D, Okay OS, Schramm KW (2006). Polycyclic aromatic hydrocarbon contamination in coastal sediments of the Izmit Bay (Marmara Sea): Case studies before and after the Izmit earthquake. Environment International.

[CR95] US EPA (United States Environmental Protection Agency). (1989). Risk Assessment Guidance for Superfund Volume I, Human Health Evaluation Manual (Part A): Interim Final. EPA/540/1 -89/002; 1989; PB90—155581, pp. 289.

[CR97] US EPA (United States Environmental Protection Agency). (2001). Integrated Risk Information System (IRIS): Benzo[a]pyrene (CAS No.50-32-8).

[CR98] US EPA (United State Environmental Protection Agency). (2003). Methods for organic chemical analysis of municipal and industrial wastewater. Method 610—Polynuclear aromatic hydrocarbons, pp. 146–152.

[CR99] US EPA (United States Environmental Protection Agency). (2004). Risk Assessment Guidance for Superfund Volume I: Human Health Evaluation Manual (Part E, Supplemental Guidance for Dermal Risk Assessment): Final. EPA/540/R/99/005 OSWER 9285.7-02EP PB99-963312, pp. 156.

[CR100] US EPA (United States Environmental Protection Agency). (2009). RAGS: Part F, Supplemental Guidance for Inhalation Risk Assessment. EPA/540/R/070/002, 2009.

[CR101] US EPA (United States Environmental Protection Agency). (2015). Regional Screening Table. Updated 2015; pp. 176. Accessed 08 Aug 2018, https://semspub.epa.gov/work/10/500011899.pdf.

[CR102] USP (United States Pharmacopoeia). (2007). The National Formulary, USP 30/NF 25, < 1225 > Validation Compendial Procedures, pp. 680–683.

[CR103] Van den Berg M, Birnbaum LS, Denison M, De Vito M, Farland W, Feeley M, Fiedler H, Hakansson H, Hanberg A, Haws L, Rose M, Safe S, Schrenk D, Tohyama C, Tritscher A, Tuomisto J, Tysklind M, Walker N, Peterson RE (2006). The 2005 World Health Organization reevaluation of human and mammalian toxic equivalency factors for dioxins and dioxin-like compounds. Toxicological Sciences.

[CR104] Viguri J, Verde J, Irabien A (2002). Environmental assessment of polycyclic aromatic hydrocarbons (PAHs) in surface sediments of the Santander Bay, Northern Spain. Chemosphere.

[CR105] Wang B, Yu G, Yu YJ, Huang J, Hu HY, Wang LS (2009). Health risk assessment of organic pollutants in Jiangsu Reach of the Huaihe River, China. Water Science and Technology.

[CR106] Wang L, Zhang S, Wang L, Zhang W, Shi X, Lu X, Li X, Li X (2018). Concentration and Risk Evaluation of Polycyclic Aromatic Hydrocarbons in Urban Soil in the Typical Semi-Arid City of Xi’an in Northwest China. International Journal of Environmental Research and Public Health.

[CR107] WDNR (Wisconsin Department of Natural Resources). (1996). Analytical Detection Limit Guidance and Laboratory Guide for Determining Method Detection Limits. 1996; PUBL-TS-056-96. Accessed 05 Oct 2017, http://dnr.wi.gov/regulations/labcert/documents/guidance/-lodguide.pdf.

[CR108] WDNR (Wisconsin Department of Natural Resources). (2003). Consensus-Based Sediment Quality Guidelines: Recommendations for Use & Application, RR-088, 2003; pp 40. Accessed 2 Aug 2018, https://dnr.wi.gov/files/PDF/pubs/rr/RR088.pdf.

[CR109] Wei H, Le Z, Shuxian L, Dan W, Xiaojun L, Lan J, Xiping M (2015). Health risk assessment of heavy metals and polycyclic aromatic hydrocarbons in soil at coke oven gas plants. Environmental Engineering and Management Journal.

[CR110] Wilson NK, Chuang JC, Lyu C (2001). Levels of persistent organic pollutants in several child day care centers. Journal of Exposure Analysis and Environmental Epidemiology.

[CR111] WSDE (Washington State Department of Ecology). (1997). Analytical methods for petroleum hydrocarbons. Department of Ecology Publications Distribution Center, Olympia, WA, 1997; Publication No. ECY 97-602, pp. 65–100.

[CR112] Yahaya A, Okoh OO, Okoh AI, Adeniji AO (2017). Occurrences of organochlorine pesticides along the course of Buffalo River in the Eastern Cape of South Africa and its health implications. International Journal of Environmental Research and Public Health.

[CR113] Yim UH, Hong SH, Shim WJ, Oh JR, Chang M (2005). Spatial-temporal distribution and characteristics of PAHs in sediments from Masan Bay. Korea. Marine Pollution Bulletin.

[CR114] Zhang W, Zhang S, Wan C, Yue D, Ye Y, Wang X (2008). Source diagnostics of polycyclic aromatic hydrocarbons in urban road runoff, dust, rain and canopy through fall. Environmental Pollution.

[CR115] Zhou JL, Hong H, Zhang Z, Maskaoui K, Chen W (2000). Multi-phase distribution of organic micropollutants in Xiamen Harbour. China. Water Research.

[CR116] Zhou JL, Maskaoui K (2003). Distribution of polycyclic aromatic hydrocarbons in water and surface sediments from Daya Bay, China. Environmental Pollution.

