# Supplementary material for: Distribution pattern and health risk assessment of polycyclic aromatic hydrocarbons in the water and sediment of Algoa Bay, South Africa
Source: Environ Geochem Health. 2018 Nov 11;41(3):1303–20. doi: 10.1007/s10653-018-0213-x (PMC6702189; doi:10.1007/s10653-018-0213-x)
Supplement: Supplementary file 1 — Supplementary material 1 (DOCX 87 kb) [file 10653_2018_213_MOESM1_ESM.docx]

| **POLYCYCLIC AROMATIC HYDROCARBON** | | | |  |  |
| --- | --- | --- | --- | --- | --- |
|  | **ALGOA BAY** | |  |  |  |
|  | **SURFACE WATER** | |  |  |  |
|  |  |  |  |  |  |
|  | **DECEMBER** | |  |  |  |
|  | **AS1** | **AS2** | **AS3** | **AS4** | **AS5** |
| Nap |  |  |  |  |  |
| Ace |  |  |  |  |  |
| Acy |  |  |  |  |  |
| Flu |  | 1.22725 | 1.671264 | 1.333513 | 4.658391 |
| Ant | 8.332624 | 2.106228 | 3.135512 |  | 1.22295 |
| Phe | 6.019653 |  | 5.555502 | 14.08284 | 4.263276 |
| Flt |  |  | 1.058431 | 0.397525 | 2.766743 |
| Pyr | 1.212136 | 1.583845 | 1.627471 | 1.354633 | 1.708633 |
| BaA | 2.072311 | 1.855999 | 1.895888 | 4.953718 | 3.4429 |
| Chry | 2.914519 | 21.22062 | 0.858313 | 7.426146 | 16.51005 |
| BbF | 7.809644 | 3.66858 | 3.382225 | 3.033776 | 4.265256 |
| BkF | 1.502396 | 7.417156 | 10.71049 | 1.887756 | 14.48053 |
| BaP | 7.043882 | 2.385953 |  | 1.60278 |  |
| DiahA | 11.72545 | 9.691242 | 13.29237 | 11.60238 | 10.0488 |
| InPy | 10.64106 | 10.00049 | 5.79556 | 11.38146 | 9.271617 |
| BghiP | 16.33077 | 4.901068 | 6.221597 | 5.66444 | 6.297553 |
| **TOTAL** | **75.60444** | **66.05842** | **55.20462** | **64.72096** | **78.9367** |
|  |  |  |  |  |  |
| 2 Ring |  |  |  |  |  |
| 3 Ring | 14.35228 | 3.333479 | 10.36228 | 15.41635 | 10.14462 |
| 4 Ring | 6.198966 | 24.66046 | 5.440103 | 14.13202 | 24.42833 |
| 5 Ring | 28.08137 | 23.16293 | 27.38508 | 18.12669 | 28.79459 |
| 6 Ring | 26.97183 | 14.90155 | 12.01716 | 17.0459 | 15.56917 |
| LMW | 14.35228 | 3.333479 | 10.36228 | 15.41635 | 10.14462 |
| HMW | 61.25217 | 62.72494 | 44.84234 | 49.30461 | 68.79208 |
| cPAHs | 60.04003 | 61.1411 | 42.15644 | 47.55245 | 64.31671 |
| LMW/HMW | 0.234315 | 0.053144 | 0.231082 | 0.312676 | 0.147468 |
|  |  |  |  |  |  |
| Phe/Ant | 0.72242 |  | 1.7718 |  | 3.486059 |
| Fla/Pyr |  |  | 0.650354 | 0.293456 | 1.619273 |
| An/Ant+Phe | 0.580579 | 1 | 0.360776 |  | 0.222913 |
| Fla/Fla+Pry |  |  | 0.394069 | 0.226877 | 0.618215 |
| Chry/BaA | 1.40641 | 11.43353 | 0.452723 | 1.499105 | 4.795392 |
| An/178 | 0.046812 | 0.011833 | 0.017615 |  | 0.006871 |
| BaA/228 | 0.009089 | 0.00814 | 0.008315 | 0.021727 | 0.0151 |
| BaA/BaA+Chry | 0.415557 | 0.080428 | 0.688362 | 0.400143 | 0.172551 |
| InPy/InPy+BghiP | 0.394525 | 0.671104 | 0.482274 | 0.667695 | 0.595511 |

|  |  |  |  |  |  |
| --- | --- | --- | --- | --- | --- |
|  |  |  |  |  |  |
|  |  |  |  |  |  |
|  |  |  |  |  |  |
|  | **FEBRUARY** | |  |  |  |
|  | **AS1** | **AS2** | **AS3** | **AS4** | **AS5** |
| Nap |  |  |  |  |  |
| Ace |  |  |  |  |  |
| Acy | 1.373332 | 1.683102 | 1.379199 | 1.161288 | 0.908913 |
| Flu | 1.351469 | 2.069202 | 3.167448 | 5.344487 | 3.68517 |
| Ant | 7.1361 | 8.634206 | 10.02835 | 14.14265 | 9.874855 |
| Phe |  |  |  |  | 15.11413 |
| Flt | 0.136667 | 0.052435 | 0.142256 | 0.093587 | 0.019857 |
| Pyr | 3.377651 | 1.847165 | 4.077182 | 2.996018 | 7.51058 |
| BaA | 1.919588 | 1.453232 | 1.227537 | 2.113818 | 1.860231 |
| Chry | 3.471933 | 5.030139 | 2.531039 | 3.592785 | 3.176228 |
| BbF | 4.309647 | 3.048552 | 1.676429 | 2.116992 |  |
| BkF | 3.690837 | 2.472322 |  | 2.336103 |  |
| BaP | 3.929137 | 3.089787 |  |  |  |
| DiahA |  |  |  |  |  |
| InPy | 4.665423 | 4.431632 |  |  |  |
| BghiP | 6.7773 | 4.970257 |  |  |  |
| **TOTAL** | **42.13908** | **38.78203** | **24.22944** | **33.89773** | **42.14997** |
|  |  |  |  |  |  |
| 2 Ring |  |  |  |  |  |
| 3 Ring | 9.860901 | 12.38651 | 14.575 | 20.64842 | 29.58307 |
| 4 Ring | 8.905838 | 8.382971 | 7.978013 | 8.796207 | 12.5669 |
| 5 Ring | 11.92962 | 8.610661 | 1.676429 | 4.453095 |  |
| 6 Ring | 11.44272 | 9.401889 |  |  |  |
| LMW | 9.860901 | 12.38651 | 14.575 | 20.64842 | 29.58307 |
| HMW | 32.27818 | 26.39552 | 9.654441 | 13.2493 | 12.5669 |
| cPAHs | 28.76387 | 24.49592 | 5.435004 | 10.1597 | 5.036459 |
| LMW/HMW | 0.305497 | 0.469266 | 1.509668 | 1.558454 | 2.354047 |
|  |  |  |  |  |  |
| Phe/Ant |  |  |  |  | 1.530567 |
| Fla/Pyr | 0.040462 | 0.028387 | 0.034891 | 0.031237 | 0.002644 |
| An/Ant+Phe | 1 | 1 | 1 | 1 | 0.395168 |
| Fla/Fla+Pry | 0.038888 | 0.027603 | 0.033714 | 0.030291 | 0.002637 |
| Chry/BaA | 1.808686 | 3.461345 | 2.061884 | 1.699666 | 1.707438 |
| An/178 | 0.04009 | 0.048507 | 0.056339 | 0.079453 | 0.055477 |
| BaA/228 | 0.008419 | 0.006374 | 0.005384 | 0.009271 | 0.008159 |
| BaA/BaA+Chry | 0.356038 | 0.224148 | 0.326596 | 0.370416 | 0.369353 |
| InPy/InPy+BghiP | 0.40772 | 0.471355 |  |  |  |

|  |  |  |  |  |  |
| --- | --- | --- | --- | --- | --- |
|  |  |  |  |  |  |
|  |  |  |  |  |  |
|  |  |  |  |  |  |
|  | **MARCH** |  |  |  |  |
|  | **AS1** | **AS2** | **AS3** | **AS4** | **AS5** |
| Nap |  |  |  |  |  |
| Ace | 8.999213 | 10.28385 | 9.842703 | 10.70823 | 9.8905 |
| Acy | 11.10844 | 10.73179 | 12.26775 | 13.09513 | 12.49405 |
| Flu | 6.15765 | 6.278981 | 6.895977 | 7.173597 | 7.034129 |
| Ant | 2.478412 | 2.330641 | 2.946234 | 2.955245 | 3.209871 |
| Phe |  |  |  |  |  |
| Flt |  |  |  |  |  |
| Pyr |  |  |  |  |  |
| BaA |  |  |  |  |  |
| Chry |  |  |  |  |  |
| BbF |  |  |  |  |  |
| BkF |  |  |  |  |  |
| BaP |  |  |  |  |  |
| DiahA |  |  |  |  |  |
| InPy |  |  |  |  |  |
| BghiP |  |  |  |  |  |
| **TOTAL** | **28.74371** | **29.62527** | **31.95266** | **33.9322** | **32.62855** |
|  |  |  |  |  |  |
| 2 Ring |  |  |  |  |  |
| 3 Ring | 28.74371 | 29.62527 | 31.95266 | 33.9322 | 32.62855 |
| 4 Ring |  |  |  |  |  |
| 5 Ring |  |  |  |  |  |
| 6 Ring |  |  |  |  |  |
| LMW | 28.74371 | 29.62527 | 31.95266 | 33.9322 | 32.62855 |
| HMW |  |  |  |  |  |
| cPAHs |  |  |  |  |  |
| LMW/HMW | |  |  |  |  |
|  |  |  |  |  |  |
| Phe/Ant |  |  |  |  |  |
| Fla/Pyr |  |  |  |  |  |
| An/Ant+Phe | 1 | 1 | 1 | 1 | 1 |
| Fla/Fla+Pry | |  |  |  |  |
| Chry/BaA |  |  |  |  |  |
| An/178 | 0.013924 | 0.013093 | 0.016552 | 0.016603 | 0.018033 |
| BaA/228 |  |  |  |  |  |
| BaA/BaA+Chry | |  |  |  |  |
| InPy/InPy+BghiP | |  |  |  |  |

|  |  |  |  |  |  |
| --- | --- | --- | --- | --- | --- |
|  |  |  |  |  |  |
|  |  |  |  |  |  |
|  |  |  |  |  |  |
|  | **MAY** |  |  |  |  |
|  | **AS1** | **AS2** | **AS3** | **AS4** | **AS5** |
| Nap | 5.263491 | 5.529606 | 4.325465 | 3.358008 | 4.30625 |
| Ace |  |  |  |  |  |
| Acy |  |  |  |  | 0.048737 |
| Flu | 1.973021 | 2.297575 | 2.949135 | 4.026 |  |
| Ant |  |  |  |  |  |
| Phe |  |  |  |  |  |
| Flt |  |  |  |  |  |
| Pyr |  |  |  |  |  |
| BaA | 0.364002 | 0.455756 | 0.329625 | 0.117011 | 0.59488 |
| Chry | 2.436924 | 0.20728 | 3.524207 | 24.66109 | 11.36914 |
| BbF | 0.36018 | 0.607176 | 0.455057 | 0.583555 | 0.928311 |
| BkF | 0.713754 | 0.330949 | 0.324294 | 0.436365 | 1.357878 |
| BaP | 1.819726 | 2.352926 | 2.091495 | 2.871108 | 0.685108 |
| DiahA |  |  |  |  |  |
| InPy | 1.575211 | 1.001986 | 0.820554 | 1.063056 | 1.370093 |
| BghiP |  |  |  |  |  |
| **TOTAL** | **14.50631** | **12.78325** | **14.81983** | **37.11619** | **20.6604** |
|  |  |  |  |  |  |
| 2 Ring | 5.263491 | 5.529606 | 4.325465 | 3.358008 | 4.30625 |
| 3 Ring | 1.973021 | 2.297575 | 2.949135 | 4.026 | 0.048737 |
| 4 Ring | 2.800926 | 0.663036 | 3.853831 | 24.7781 | 11.96402 |
| 5 Ring | 2.89366 | 3.291051 | 2.870846 | 3.891028 | 2.971297 |
| 6 Ring | 1.575211 | 1.001986 | 0.820554 | 1.063056 | 1.370093 |
| LMW | 7.236513 | 7.827181 | 7.2746 | 7.384008 | 4.354987 |
| HMW | 7.269798 | 4.956073 | 7.545231 | 29.73218 | 16.30541 |
| cPAHs | 7.269798 | 4.956073 | 7.545231 | 29.73218 | 16.30541 |
| LMW/HMW | 0.995421 | 1.579311 | 0.964132 | 0.248351 | 0.267088 |
|  |  |  |  |  |  |
| Phe/Ant |  |  |  |  |  |
| Fla/Pyr |  |  |  |  |  |
| An/Ant+Phe | |  |  |  |  |
| Fla/Fla+Pry | |  |  |  |  |
| Chry/BaA | 6.694814 | 0.454805 | 10.69158 | 210.7584 | 19.11166 |
| An/178 |  |  |  |  |  |
| BaA/228 | 0.001596 | 0.001999 | 0.001446 | 0.000513 | 0.002609 |
| BaA/BaA+Chry | 0.129958 | 0.687377 | 0.085532 | 0.004722 | 0.049722 |
| InPy/InPy+BghiP | 1 | 1 | 1 | 1 | 1 |

|  |  |  |  |  |  |
| --- | --- | --- | --- | --- | --- |
|  |  |  |  |  |  |
|  |  |  |  |  |  |
|  |  |  |  |  |  |
|  | **JUNE** |  |  |  |  |
|  | **AS1** | **AS2** | **AS3** | **AS4** | **AS5** |
| Nap | 3.788917 | 4.306492 | 3.2362 | 4.122228 | 3.939968 |
| Ace |  |  |  |  |  |
| Acy | 0.882531 | 1.227133 | 0.316057 | 0.604263 | 0.081311 |
| Flu | 3.445201 | 3.410988 | 2.400032 | 2.511317 | 3.603093 |
| Ant |  |  |  |  |  |
| Phe |  |  |  |  |  |
| Flt |  |  |  |  |  |
| Pyr |  | 0.640805 |  |  |  |
| BaA | 0.758195 | 0.924433 | 1.01548 | 1.022573 | 0.808216 |
| Chry | 2.168583 | 0.593074 | 1.811182 | 1.121414 | 1.050227 |
| BbF | 0.972718 | 1.386333 | 0.821617 | 1.166811 | 1.064999 |
| BkF | 1.824751 | 1.506569 | 0.124492 | 9.219639 | 0.062418 |
| BaP | 1.065494 | 1.408722 | 0.869604 | 1.540723 | 1.09568 |
| DiahA |  |  | 1.652538 | 2.102224 |  |
| InPy | 1.380333 | 1.58504 | 1.618832 | 1.705839 | 1.259368 |
| BghiP |  | 0.661132 |  | 1.187187 |  |
| **TOTAL** | **16.28672** | **17.65072** | **13.86603** | **26.30422** | **12.96528** |
|  |  |  |  |  |  |
| 2 Ring | 3.788917 | 4.306492 | 3.2362 | 4.122228 | 3.939968 |
| 3 Ring | 4.327733 | 4.638121 | 2.716089 | 3.11558 | 3.684404 |
| 4 Ring | 2.926777 | 2.158313 | 2.826662 | 2.143987 | 1.858443 |
| 5 Ring | 3.862963 | 4.301624 | 3.468251 | 14.0294 | 2.223096 |
| 6 Ring | 1.380333 | 2.246172 | 1.618832 | 2.893026 | 1.259368 |
| LMW | 8.116649 | 8.944613 | 5.952289 | 7.237807 | 7.624372 |
| HMW | 8.170074 | 8.706109 | 7.913745 | 19.06641 | 5.340907 |
| cPAHs | 8.170074 | 8.065304 | 7.913745 | 19.06641 | 5.340907 |
| LMW/HMW | 0.993461 | 1.027395 | 0.752146 | 0.37961 | 1.427543 |
|  |  |  |  |  |  |
| Phe/Ant |  |  |  |  |  |
| Fla/Pyr |  |  |  |  |  |
| An/Ant+Phe | |  |  |  |  |
| Fla/Fla+Pry | |  |  |  |  |
| Chry/BaA | 2.860192 | 0.641554 | 1.783573 | 1.096659 | 1.299439 |
| An/178 |  |  |  |  |  |
| BaA/228 | 0.003325 | 0.004055 | 0.004454 | 0.004485 | 0.003545 |
| BaA/BaA+Chry | 0.259054 | 0.609179 | 0.35925 | 0.476949 | 0.434889 |
| InPy/InPy+BghiP | 1 | 0.705663 | 1 | 0.589638 | 1 |

|  |  |  |  |  |  |
| --- | --- | --- | --- | --- | --- |
|  |  |  |  |  |  |
|  |  |  |  |  |  |
|  |  |  |  |  |  |
|  |  |  |  |  |  |
|  | **MIN** | **MAX** | **AVERAGE** | **SD** | **SE** |
| Nap | 3.236200306 | 5.529606 | 4.217663 | 0.731409 | 0.146282 |
| Ace | 8.999213296 | 10.70823 | 9.9449 | 0.633501 | 0.1267 |
| Acy | 0.048736648 | 13.09513 | 4.335189 | 5.338556 | 1.067711 |
| Flu | 1.2272502 | 7.173597 | 3.681082 | 1.940851 | 0.38817 |
| Ant | 1.22295 | 14.14265 | 5.609563 | 3.995335 | 0.799067 |
| Phe | 4.2632757 | 15.11413 | 9.00708 | 5.157548 | 1.03151 |
| Flt | 0.019857365 | 2.766743 | 0.583438 | 0.945727 | 0.189145 |
| Pyr | 0.640805203 | 7.51058 | 2.539647 | 1.940943 | 0.388189 |
| BaA | 0.117011151 | 4.953718 | 1.45927 | 1.154973 | 0.230995 |
| Chry | 0.207280009 | 24.66109 | 5.783744 | 7.076738 | 1.415348 |
| BbF | 0.360180053 | 7.809644 | 2.192519 | 1.901088 | 0.380218 |
| BkF | 0.062417741 | 14.48053 | 3.355484 | 4.208812 | 0.841762 |
| BaP | 0.685108224 | 7.043882 | 2.256808 | 1.600225 | 0.320045 |
| DiahA | 1.652538226 | 13.29237 | 8.587856 | 4.735999 | 0.9472 |
| InPy | 0.820554052 | 11.38146 | 4.092209 | 3.854798 | 0.77096 |
| BghiP | 0.661132154 | 16.33077 | 5.890145 | 4.491734 | 0.898347 |
| **TOTAL** | **12.78325375** | **78.9367** | **73.5366** | **19.52825** | **9.941647** |
|  |  |  |  |  |  |
|  |  |  |  |  |  |
|  |  |  |  |  |  |
|  |  |  |  |  |  |
|  |  |  |  |  |  |
| LMW | 3.3334785 | 65.76334 | 36.79548 | 10.08552 | 2.017103 |
| HMW | 4.956072888 | 110.2308 | 36.74112 | 21.27886 | 4.255771 |
| cPAHs | 4.956072888 | 64.31671 | 27.72789 | 20.81803 | 4.163606 |
|  | LMW/HMW | **0.632174** | **1.001479** | **0.864257** | **0.624773** |

|  |  |  |  |  |  |
| --- | --- | --- | --- | --- | --- |
|  |  |  |  |  |  |
|  |  |  |  |  |  |
|  |  |  |  |  |  |
|  |  |  |  |  |  |
|  | **MIN** | **MAX** | **AVERAGE** | **SD** | **SE** |
| Nap | 3.236200306 | 5.529606 | 4.217663 | 0.731409 | 0.146282 |
| Ace | 8.999213296 | 10.70823 | 9.9449 | 0.633501 | 0.1267 |
| Acy | 0.048736648 | 13.09513 | 4.335189 | 5.338556 | 1.067711 |
| Flu | 1.2272502 | 7.173597 | 3.681082 | 1.940851 | 0.38817 |
| Ant | 1.22295 | 14.14265 | 5.609563 | 3.995335 | 0.799067 |
| Phe | 4.2632757 | 15.11413 | 9.00708 | 5.157548 | 1.03151 |
| Flt | 0.019857365 | 2.766743 | 0.583438 | 0.945727 | 0.189145 |
| Pyr | 0.640805203 | 7.51058 | 2.539647 | 1.940943 | 0.388189 |
| BaA | 0.117011151 | 4.953718 | 1.45927 | 1.154973 | 0.230995 |
| Chry | 0.207280009 | 24.66109 | 5.783744 | 7.076738 | 1.415348 |
| BbF | 0.360180053 | 7.809644 | 2.192519 | 1.901088 | 0.380218 |
| BkF | 0.062417741 | 14.48053 | 3.355484 | 4.208812 | 0.841762 |
| BaP | 0.685108224 | 7.043882 | 2.256808 | 1.600225 | 0.320045 |
| DiahA | 1.652538226 | 13.29237 | 8.587856 | 4.735999 | 0.9472 |
| InPy | 0.820554052 | 11.38146 | 4.092209 | 3.854798 | 0.77096 |
| BghiP | 0.661132154 | 16.33077 | 5.890145 | 4.491734 | 0.898347 |
| **TOTAL** | **12.78325375** | **78.9367** | **73.5366** | **19.52825** | **9.941647** |

|  | **BOTTOM WATER** | |  |  |  |
| --- | --- | --- | --- | --- | --- |
|  | **DECEMBER** | |  |  |  |
|  | **AB1** | **AB2** | **AB3** | **AB4** | **AB5** |
| Nap |  |  |  |  |  |
| Ace |  |  |  |  |  |
| Acy |  |  |  |  |  |
| Flu |  |  |  |  |  |
| Ant | 8.481878 | 3.570035 | 14.89251 | 3.119566 | 2.515123 |
| Phe | 2.807145 | 6.35584 | 3.792835 | 8.540553 | 13.0203 |
| Flt |  |  | 0.615007 |  |  |
| Pyr | 2.361589 | 1.463117 | 1.370674 | 2.532275 | 2.440914 |
| BaA | 2.051649 | 4.862557 | 1.394085 |  | 22.80754 |
| Chry | 12.37555 | 9.753489 | 14.26475 | 17.66833 | 7.673188 |
| BbF | 7.908325 | 5.008476 | 5.601743 | 2.447756 |  |
| BkF | 7.863323 | 6.682343 | 7.553348 | 11.6491 | 8.043176 |
| BaP | 3.937005 | 2.876576 | 5.683207 | 0.42502 |  |
| DiahA | 20.85913 | 13.9173 | 10.80566 | 13.93268 | 3.876333 |
| InPy | 5.600551 | 7.963162 | 15.11154 | 8.874986 | 9.705912 |
| BghiP | 9.96432 | 7.774451 | 9.425286 | 7.516857 | 6.324564 |
| **TOTAL** | **84.21046** | **70.22735** | **90.51065** | **76.70712** | **76.40705** |
|  |  |  |  |  |  |
| 2 Ring |  |  |  |  |  |
| 3 Ring | 11.28902 | 9.925875 | 18.68535 | 11.66012 | 15.53542 |
| 4 Ring | 16.78879 | 16.07916 | 17.64452 | 20.2006 | 32.92164 |
| 5 Ring | 40.56778 | 28.48469 | 29.64395 | 28.45456 | 11.91951 |
| 6 Ring | 15.56487 | 15.73761 | 24.53683 | 16.39184 | 16.03048 |
| LMW | 11.28902 | 9.925875 | 18.68535 | 11.66012 | 15.53542 |
| HMW | 72.92144 | 60.30147 | 71.8253 | 65.047 | 60.87163 |
| cPAHs | 70.55985 | 58.83835 | 69.83962 | 62.51473 | 58.43071 |
| LMW/HMW | 0.154811 | 0.164604 | 0.26015 | 0.179257 | 0.255216 |
|  |  |  |  |  |  |
| Phe/Ant | 0.330958 | 1.78033 | 0.254681 | 2.737738 | 5.176805 |
| Fla/Pyr |  |  | 0.448689 |  |  |
| An/Ant+Phe | 0.751339 | 0.35967 | 0.797016 | 0.267541 | 0.161896 |
| Fla/Fla+Pry |  |  | 0.309721 |  |  |
| Chry/BaA | 6.032 | 2.005835 | 10.23234 |  | 0.336432 |
| An/178 | 0.047651 | 0.020056 | 0.083666 | 0.017526 | 0.01413 |
| BaA/228 | 0.008998 | 0.021327 | 0.006114 |  | 0.100033 |
| BaA/BaA+Chry | 0.142207 | 0.332686 | 0.089029 |  | 0.748261 |
| InPy/InPy+BghiP | 0.35982 | 0.505996 | 0.615872 | 0.541427 | 0.605466 |

|  |  |  |  |  |  |
| --- | --- | --- | --- | --- | --- |
|  | **FEBRUARY** | |  |  |  |
|  | **AB1** | **AB2** | **AB3** | **AB4** | **AB5** |
| Nap |  |  | 7.776982 |  |  |
| Ace |  |  |  |  |  |
| Acy | 0.527089 | 0.958203 | 1.148032 | 0.923644 | 0.742685 |
| Flu | 3.168458 | 3.297935 | 3.413265 | 6.466837 | 3.439012 |
| Ant | 9.519597 | 13.86281 | 13.37403 | 10.2223 | 9.28367 |
| Phe | 14.26442 |  |  | 2.639505 | 12.34102 |
| Flt | 0.139299 | 0.015291 | 0.024334 |  |  |
| Pyr | 0.775201 | 0.810789 | 3.913801 | 6.629288 | 6.325832 |
| BaA | 5.29983 | 3.284269 | 4.961977 | 7.274071 | 3.171306 |
| Chry | 3.816107 | 4.152971 | 3.622132 | 3.20287 | 2.859332 |
| BbF | 6.526599 | 3.693264 |  |  | 2.479349 |
| BkF | 2.716944 | 3.142259 |  |  |  |
| BaP | 8.520596 | 5.361961 | 4.377548 |  |  |
| DiahA | 14.31301 |  |  |  |  |
| InPy | 6.003809 | 6.315394 |  |  |  |
| BghiP | 12.26121 | 16.72475 |  |  |  |
| **TOTAL** | **87.85217** | **61.6199** | **42.61211** | **37.35851** | **40.64221** |
|  |  |  |  |  |  |
| 2 Ring |  |  | 7.776982 |  |  |
| 3 Ring | 27.47956 | 18.11895 | 17.93533 | 20.25228 | 25.80639 |
| 4 Ring | 10.03044 | 8.26332 | 12.52224 | 17.10623 | 12.35647 |
| 5 Ring | 32.07715 | 12.19748 | 4.377548 |  | 2.479349 |
| 6 Ring | 18.26502 | 23.04015 |  |  |  |
| LMW | 27.47956 | 18.11895 | 25.71231 | 20.25228 | 25.80639 |
| HMW | 60.3726 | 43.50095 | 16.89979 | 17.10623 | 14.83582 |
| cPAHs | 59.4581 | 42.67487 | 12.96166 | 10.47694 | 8.509986 |
| LMW/HMW | 0.455166 | 0.416519 | 1.521457 | 1.183913 | 1.739465 |
|  |  |  |  |  |  |
| Phe/Ant | 1.498427 |  |  | 0.258211 | 1.329326 |
| Fla/Pyr | 0.179694 | 0.01886 | 0.006218 |  |  |
| An/Ant+Phe | 0.400252 | 1 | 1 | 0.79478 | 0.429309 |
| Fla/Fla+Pry | 0.152322 | 0.018511 | 0.006179 |  |  |
| Chry/BaA | 0.720043 | 1.264504 | 0.729978 | 0.440313 | 0.901626 |
| An/178 | 0.053481 | 0.077881 | 0.075135 | 0.057429 | 0.052155 |
| BaA/228 | 0.023245 | 0.014405 | 0.021763 | 0.031904 | 0.013909 |
| BaA/BaA+Chry | 0.581381 | 0.441598 | 0.578042 | 0.694293 | 0.525866 |
| InPy/InPy+BghiP | 0.328705 | 0.274104 |  |  |  |

|  |  |  |  |  |  |
| --- | --- | --- | --- | --- | --- |
|  | **MARCH** |  |  |  |  |
|  | **AB1** | **AB2** | **AB3** | **AB4** | **AB5** |
| Nap |  |  |  |  |  |
| Ace | 9.700808 | 8.760695 | 10.03491 | 11.41755 | 14.48705 |
| Acy | 12.63765 | 11.06596 | 12.01625 | 14.13359 | 18.93544 |
| Flu | 7.355431 | 6.279331 | 6.590367 | 8.205471 | 12.03768 |
| Ant | 3.723997 | 3.013445 | 2.534343 | 3.752966 | 7.518499 |
| Phe |  |  |  |  |  |
| Flt |  |  |  |  |  |
| Pyr |  |  |  |  |  |
| BaA |  |  |  |  |  |
| Chry |  |  |  |  |  |
| BbF |  |  |  |  |  |
| BkF |  |  |  |  |  |
| BaP |  |  |  |  |  |
| DiahA |  |  |  |  |  |
| InPy |  |  |  |  |  |
| BghiP |  |  |  |  |  |
| **TOTAL** | **33.41788** | **29.11943** | **31.17587** | **37.50958** | **52.97866** |
|  |  |  |  |  |  |
| 2 Ring |  |  |  |  |  |
| 3 Ring | 33.41788 | 29.11943 | 31.17587 | 37.50958 | 52.97866 |
| 4 Ring |  |  |  |  |  |
| 5 Ring |  |  |  |  |  |
| 6 Ring |  |  |  |  |  |
| LMW | 33.41788 | 29.11943 | 31.17587 | 37.50958 | 52.97866 |
| HMW |  |  |  |  |  |
| cPAHs |  |  |  |  |  |
| LMW/HMW | |  |  |  |  |
|  |  |  |  |  |  |
| Phe/Ant |  |  |  |  |  |
| Fla/Pyr |  |  |  |  |  |
| An/Ant+Phe | 1 | 1 | 1 | 1 | 1 |
| Fla/Fla+Pry | |  |  |  |  |
| Chry/BaA |  |  |  |  |  |
| An/178 | 0.020921 | 0.016929 | 0.014238 | 0.021084 | 0.042239 |
| BaA/228 |  |  |  |  |  |
| BaA/BaA+Chry | |  |  |  |  |
| InPy/InPy+BghiP | |  |  |  |  |

|  |  |  |  |  |  |
| --- | --- | --- | --- | --- | --- |
|  | **MAY** |  |  |  |  |
|  | **AB1** | **AB2** | **AB3** | **AB4** | **AB5** |
| Nap | 11.05438 |  |  | 4.408 | 4.530336 |
| Ace |  | 0.625726 | 1.439818 |  |  |
| Acy | 0.280413 | 0.19709 | 1.025206 | 0.050159 |  |
| Flu |  | 2.98814 | 9.153728 |  | 3.983095 |
| Ant |  |  | 0.548376 |  |  |
| Phe |  |  |  |  |  |
| Flt |  |  | 0.05359 |  |  |
| Pyr | 0.357978 | 0.229547 | 0.106331 |  |  |
| BaA | 3.059509 | 4.412238 | 5.119195 | 0.494344 | 0.399769 |
| Chry | 5.812767 | 1.351788 | 2.217107 | 5.487415 | 1.094788 |
| BbF | 2.496284 | 1.230855 | 1.886745 | 1.14494 | 1.281652 |
| BkF | 0.316483 | 3.291036 | 7.831592 | 0.28884 | 0.798233 |
| BaP | 0.700855 | 1.139574 | 1.866527 | 0.916188 | 1.181881 |
| DiahA | 2.939712 |  |  |  |  |
| InPy | 3.674957 |  |  | 1.93566 | 3.068758 |
| BghiP | 2.374233 |  |  |  | 1.706311 |
| **TOTAL** | **33.06757** | **15.46599** | **31.24822** | **14.72554** | **18.04482** |
|  |  |  |  |  |  |
| 2 Ring | 11.05438 |  |  | 4.408 | 4.530336 |
| 3 Ring | 0.280413 | 3.810955 | 12.16713 | 0.050159 | 3.983095 |
| 4 Ring | 9.230254 | 5.993574 | 7.496224 | 5.981758 | 1.494557 |
| 5 Ring | 6.453335 | 5.661465 | 11.58486 | 2.349968 | 3.261767 |
| 6 Ring | 6.049189 |  |  | 1.93566 | 4.775069 |
| LMW | 11.33479 | 3.810955 | 12.16713 | 4.458159 | 8.51343 |
| HMW | 21.73278 | 11.65504 | 19.08109 | 10.26739 | 9.531392 |
| cPAHs | 21.3748 | 11.42549 | 18.92117 | 10.26739 | 9.531392 |
| LMW/HMW | 0.521553 | 0.326979 | 0.637654 | 0.434206 | 0.893199 |
|  |  |  |  |  |  |
| Phe/Ant |  |  |  |  |  |
| Fla/Pyr |  |  | 0.503994 |  |  |
| An/Ant+Phe | |  | 1 |  |  |
| Fla/Fla+Pry | |  | 0.335104 |  |  |
| Chry/BaA | 1.899902 | 0.306372 | 0.433097 | 11.1004 | 2.738554 |
| An/178 |  |  | 0.003081 |  |  |
| BaA/228 | 0.013419 | 0.019352 | 0.022453 | 0.002168 | 0.001753 |
| BaA/BaA+Chry | 0.344839 | 0.765478 | 0.69779 | 0.082642 | 0.267483 |
| InPy/InPy+BghiP | 0.607512 |  |  | 1 | 0.642663 |

|  |  |  |  |  |  |
| --- | --- | --- | --- | --- | --- |
|  | **JUNE** |  |  |  |  |
|  | **AB1** | **AB2** | **AB3** | **AB4** | **AB5** |
| Nap | 4.03094 | 3.728028 |  | 3.792876 |  |
| Ace |  |  | 0.111841 |  |  |
| Acy | 0.239603 | 0.123581 | 0.021114 |  |  |
| Flu | 3.232107 | 3.028019 | 0.189961 | 3.331308 | 3.260203 |
| Ant |  |  |  |  |  |
| Phe |  |  |  |  |  |
| Flt |  |  |  |  |  |
| Pyr | 0.115999 |  |  |  |  |
| BaA | 0.397993 | 0.73499 | 0.100625 | 0.466842 | 0.247628 |
| Chry | 3.827994 | 5.37603 | 0.13907 | 10.22798 | 2.278468 |
| BbF | 0.719619 | 1.074909 | 0.169741 | 0.843179 | 0.407545 |
| BkF | 0.08988 | 1.50511 |  | 0.083927 |  |
| BaP | 0.737206 | 0.594698 | 0.465691 | 0.938972 | 2.078214 |
| DiahA |  | 0.6415 |  |  |  |
| InPy | 1.419858 | 5.064621 |  | 1.269744 | 2.815518 |
| BghiP |  | 0.950861 |  |  |  |
| **TOTAL** | **14.8112** | **22.82235** | **1.198044** | **20.95483** | **11.08758** |
|  |  |  |  |  |  |
| 2 Ring | 4.03094 | 3.728028 |  | 3.792876 |  |
| 3 Ring | 3.471711 | 3.1516 | 0.322916 | 3.331308 | 3.260203 |
| 4 Ring | 4.341985 | 6.111019 | 0.239696 | 10.69482 | 2.526096 |
| 5 Ring | 1.546705 | 3.816216 | 0.635432 | 1.866078 | 2.485759 |
| 6 Ring | 1.419858 | 6.015482 |  | 1.269744 | 2.815518 |
| LMW | 7.502651 | 6.879628 | 0.322916 | 7.124185 | 3.260203 |
| HMW | 7.308548 | 15.94272 | 0.875128 | 13.83064 | 7.827373 |
| cPAHs | 7.19255 | 15.94272 | 0.875128 | 13.83064 | 7.827373 |
| LMW/HMW | 1.026558 | 0.431522 | 0.368993 | 0.515102 | 0.416513 |
|  |  |  |  |  |  |
| Phe/Ant |  |  |  |  |  |
| Fla/Pyr |  |  |  |  |  |
| An/Ant+Phe | |  |  |  |  |
| Fla/Fla+Pry | |  |  |  |  |
| Chry/BaA | 9.618255 | 7.314426 | 1.382059 | 21.90884 | 9.201159 |
| An/178 |  |  |  |  |  |
| BaA/228 | 0.001746 | 0.003224 | 0.000441 | 0.002048 | 0.001086 |
| BaA/BaA+Chry | 0.094177 | 0.120273 | 0.419805 | 0.043651 | 0.098028 |
| InPy/InPy+BghiP | 1 | 0.841931 |  | 1 | 1 |

|  |  |  |  |  |  |
| --- | --- | --- | --- | --- | --- |
|  |  |  |  |  |  |
|  | **MIN** | **MAX** | **AVERAGE** | **SD** | **SE** |
| Nap | 3.728027946 | 11.05438 | 5.617363 | 2.777419 | 0.555484 |
| Ace | 0.11184084 | 14.48705 | 7.0723 | 5.530792 | 1.106158 |
| Acy | 0.02111448 | 18.93544 | 4.413276 | 6.417352 | 1.28347 |
| Flu | 0.1899605 | 12.03768 | 4.967797 | 2.881524 | 0.576305 |
| Ant | 0.5483758 | 14.89251 | 6.870822 | 4.617597 | 0.923519 |
| Phe | 2.63950497 | 14.26442 | 7.970202 | 4.776132 | 0.955226 |
| Flt | 0.015291279 | 0.615007 | 0.169504 | 0.253809 | 0.050762 |
| Pyr | 0.10633146 | 6.629288 | 2.102381 | 2.163194 | 0.432639 |
| BaA | 0.10062548 | 22.80754 | 3.712654 | 5.115232 | 1.023046 |
| Chry | 0.1390704 | 17.66833 | 5.860106 | 4.727197 | 0.945439 |
| BbF | 0.1697412 | 7.908325 | 2.642411 | 2.311933 | 0.462387 |
| BkF | 0.083927049 | 11.6491 | 4.123706 | 3.792663 | 0.758533 |
| BaP | 0.42502 | 8.520596 | 2.458925 | 2.342617 | 0.468523 |
| DiahA | 0.641500322 | 20.85913 | 10.16067 | 6.99699 | 1.399398 |
| InPy | 1.269743676 | 15.11154 | 5.630319 | 3.844127 | 0.768825 |
| BghiP | 0.9508606 | 16.72475 | 7.502284 | 4.963573 | 0.992715 |
|  | **1.198044** | **90.51065** | **81.27472** | **25.82761** | **12.70243** |

|  | **SEDIMENT** | |  |  |  |
| --- | --- | --- | --- | --- | --- |
|  | **DECEMBER** | |  |  |  |
|  | **A1** | **A2** | **A3** | **A4** | **A5** |
| Nap |  |  |  |  |  |
| Ace |  |  |  |  |  |
| Acy |  |  |  | 263.7116 |  |
| Flu | 263.9975 | 81.66615 | 165.9511 | 124.4034 |  |
| Ant | 902.154 | 135.6234 | 127.8783 | 498.7407 |  |
| Phe | 126.537 | 130.1361 | 86.44944 | 238.5176 |  |
| Flt |  |  |  |  |  |
| Pyr | 140.0651 | 77.03055 | 80.47252 | 184.0701 |  |
| BaA | 666.507 | 919.0215 | 865.4991 | 381.5753 |  |
| Chry | 254.1146 | 106.434 | 440.6794 | 271.7422 |  |
| BbF | 336.105 | 226.8842 | 148.8534 | 110.6389 |  |
| BkF | 276.9569 | 81.9564 | 108.6487 | 1006.837 |  |
| BaP | 57.54666 | 143.5893 | 37.99191 | 65.19698 |  |
| DiahA | 232.1512 | 136.0796 | 48.14072 | 960.6856 |  |
| InPy | 278.3266 | 286.8615 | 275.0939 | 319.0332 |  |
| BghiP | 364.3279 | 496.683 | 581.2722 | 397.7308 |  |
| **TOTAL** | **3898.789** | **2821.966** | **2966.931** | **4822.883** |  |
|  |  |  |  |  |  |
| 2 Ring |  |  |  |  |  |
| 3 Ring | 1292.688 | 347.4257 | 380.2788 | 1125.373 |  |
| 4 Ring | 1060.687 | 1102.486 | 1386.651 | 837.3876 |  |
| 5 Ring | 902.7598 | 588.5095 | 343.6348 | 2143.358 |  |
| 6 Ring | 642.6545 | 783.5445 | 856.3661 | 716.764 |  |
| LMW | 1292.688 | 347.4257 | 380.2788 | 1125.373 |  |
| HMW | 2606.101 | 2474.54 | 2586.652 | 3697.51 |  |
| cPAHs | 2466.036 | 2397.509 | 2506.179 | 3513.44 |  |
| LMW/HMW | 0.496024 | 0.1404 | 0.147016 | 0.30436 |  |
|  |  |  |  |  |  |
| Phe/Ant | 0.140261 | 0.95954 | 0.676029 | 0.47824 |  |
| Fla/Pyr |  |  |  |  |  |
| An/Ant+Phe | 0.876992 | 0.510324 | 0.596648 | 0.67648 |  |
| Fla/Fla+Pry |  |  |  |  |  |
| Chry/BaA | 0.381263 | 0.115812 | 0.509162 | 0.712159 |  |
| An/178 | 5.068281 | 0.761929 | 0.718417 | 2.801914 |  |
| BaA/228 | 2.923276 | 4.030796 | 3.796049 | 1.673576 |  |
| BaA/BaA+Chry | 0.723975 | 0.896208 | 0.662619 | 0.584058 |  |
| InPy/InPy+BghiP | 0.433089 | 0.366107 | 0.321234 | 0.445102 |  |

|  |  |  |  |  |  |
| --- | --- | --- | --- | --- | --- |
|  | **FEBRUARY** | |  |  |  |
|  | **A1** | **A2** | **A3** | **A4** | **A5** |
| Nap |  |  |  |  |  |
| Ace |  |  |  |  |  |
| Acy |  |  | 41.99253 |  | 40.80499 |
| Flu | 761.01 | 801.9082 | 924.839 | 773.1102 | 943.5668 |
| Ant | 445.0139 | 589.7178 | 785.7092 | 568.0715 | 618.7585 |
| Phe |  |  | 158.4206 |  |  |
| Flt |  |  | 5.7306 |  |  |
| Pyr | 27.70346 | 33.79107 | 39.98934 | 31.42409 | 34.12796 |
| BaA | 106.8182 | 133.9278 | 171.9075 | 118.383 | 125.8245 |
| Chry | 163.8797 | 214.3774 | 213.7816 | 210.8475 | 218.1376 |
| BbF | 804.2827 | 524.5183 | 422.5773 | 324.2606 | 248.6026 |
| BkF | 118.9257 | 150.7534 | 135.3374 | 130.9216 | 128.969 |
| BaP | 244.142 | 245.7259 | 260.9786 | 206.3962 | 436.4973 |
| DiahA |  |  |  |  |  |
| InPy |  |  |  |  |  |
| BghiP | 412.8283 |  |  |  |  |
| **TOTAL** | **3084.604** | **2694.72** | **3161.264** | **2363.415** | **2795.289** |
|  |  |  |  |  |  |
| 2 Ring |  |  |  |  |  |
| 3 Ring | 1206.024 | 1391.626 | 1910.961 | 1341.182 | 1603.13 |
| 4 Ring | 298.4013 | 382.0963 | 431.409 | 360.6546 | 378.09 |
| 5 Ring | 1167.35 | 920.9976 | 818.8934 | 661.5784 | 814.069 |
| 6 Ring | 412.8283 |  |  |  |  |
| LMW | 1206.024 | 1391.626 | 1910.961 | 1341.182 | 1603.13 |
| HMW | 1878.58 | 1303.094 | 1250.302 | 1022.233 | 1192.159 |
| cPAHs | 1850.877 | 1269.303 | 1204.582 | 990.8089 | 1158.031 |
| LMW/HMW | 0.641987 | 1.06794 | 1.528399 | 1.312012 | 1.344729 |
|  |  |  |  |  |  |
| Phe/Ant |  |  | 0.201628 |  |  |
| Fla/Pyr |  |  | 0.143303 |  |  |
| An/Ant+Phe | 1 | 1 | 0.832205 | 1 | 1 |
| Fla/Fla+Pry | |  | 0.125341 |  |  |
| Chry/BaA | 1.534193 | 1.600693 | 1.243585 | 1.781063 | 1.733666 |
| An/178 | 2.500078 | 3.313021 | 4.414097 | 3.191413 | 3.476171 |
| BaA/228 | 0.468501 | 0.587403 | 0.75398 | 0.519224 | 0.551862 |
| BaA/BaA+Chry | 0.394603 | 0.384513 | 0.445715 | 0.359575 | 0.365809 |
| InPy/InPy+BghiP | |  |  |  |  |

|  |  |  |  |  |  |
| --- | --- | --- | --- | --- | --- |
|  | **MARCH** |  |  |  |  |
|  | **A1** | **A2** | **A3** | **A4** | **A5** |
| Nap |  |  |  |  | 268.7927 |
| Ace | 72.22841 | 23.90032 | 70.5108 | 73.2093 | 21.19469 |
| Acy | 48.03333 | 45.66192 | 42.86025 | 39.95715 | 12.51855 |
| Flu | 32.06863 | 32.6616 | 34.7307 | 25.521 | 405.174 |
| Ant | 187.1173 | 190.176 | 132.5327 | 61.2282 | 52.51037 |
| Phe | 65.86769 | 39.7168 | 47.34525 | 24.12405 | 59.6618 |
| Flt | 591.4946 | 631.376 | 643.428 | 618.1275 | 23.25796 |
| Pyr | 57.04894 | 127.4979 | 125.9117 | 138.0887 | 21.7074 |
| BaA | 48.86344 | 47.1848 | 56.63355 | 45.7641 | 1099.761 |
| Chry | 243.9789 | 175.8368 | 173.1885 | 119.4335 | 136.2317 |
| BbF |  |  | 47.1234 | 43.82475 | 105.449 |
| BkF |  |  |  |  |  |
| BaP |  |  |  |  | 57.9491 |
| DiahA |  |  |  |  | 130.889 |
| InPy |  |  |  |  | 73.24757 |
| BghiP |  |  |  |  |  |
| **TOTAL** | **1346.701** | **1314.012** | **1374.265** | **1189.278** | **2468.345** |
|  |  |  |  |  |  |
| 2 Ring |  |  |  |  | 268.7927 |
| 3 Ring | 405.3154 | 332.1166 | 327.9797 | 224.0397 | 551.0594 |
| 4 Ring | 941.3859 | 981.8955 | 999.1617 | 921.4137 | 1280.958 |
| 5 Ring |  |  | 47.1234 | 43.82475 | 294.2871 |
| 6 Ring |  |  |  |  | 73.24757 |
| LMW | 405.3154 | 332.1166 | 327.9797 | 224.0397 | 819.8521 |
| HMW | 941.3859 | 981.8955 | 1046.285 | 965.2385 | 1648.493 |
| cPAHs | 292.8423 | 223.0216 | 276.9455 | 209.0223 | 1603.528 |
| LMW/HMW | 0.430552 | 0.33824 | 0.313471 | 0.232108 | 0.497334 |
|  |  |  |  |  |  |
| Phe/Ant | 0.352013 | 0.208842 | 0.357235 | 0.394002 | 1.136191 |
| Fla/Pyr | 10.3682 | 4.952049 | 5.110155 | 4.476309 | 1.07143 |
| An/Ant+Phe | 0.739638 | 0.827238 | 0.736792 | 0.717359 | 0.468123 |
| Fla/Fla+Pry | 0.912035 | 0.831991 | 0.836338 | 0.817395 | 0.517242 |
| Chry/BaA | 4.993077 | 3.726556 | 3.058055 | 2.609763 | 0.123874 |
| An/178 | 1.051221 | 1.068404 | 0.744565 | 0.343979 | 0.295002 |
| BaA/228 | 0.214313 | 0.206951 | 0.248393 | 0.20072 | 4.823514 |
| BaA/BaA+Chry | 0.166859 | 0.211571 | 0.246423 | 0.277027 | 0.88978 |
| InPy/InPy+BghiP | |  |  |  | 1 |

|  |  |  |  |  |  |
| --- | --- | --- | --- | --- | --- |
|  | **MAY** |  |  |  |  |
|  | **A1** | **A2** | **A3** | **A4** | **A5** |
| Nap |  | 132.8935 | 168.9297 | 145.8916 | 187.6999 |
| Ace |  |  |  |  |  |
| Acy |  | 0.140904 | 9.046602 |  |  |
| Flu | 1085.339 | 917.8286 | 998.2637 | 903.7953 | 869.0563 |
| Ant |  |  |  |  |  |
| Phe |  |  |  |  |  |
| Flt |  |  |  |  |  |
| Pyr |  |  |  |  |  |
| BaA | 177.3662 | 44.45459 | 36.23779 | 21.56632 | 29.65126 |
| Chry | 230.1217 |  | 24.04983 |  | 28.00117 |
| BbF | 346.0501 | 31.59789 | 37.56453 | 28.97087 | 49.13488 |
| BkF | 30.23038 | 33.27444 | 28.7912 | 30.5905 | 12.81048 |
| BaP | 366.5542 | 34.35218 | 57.09369 | 37.45891 | 34 |
| DiahA |  |  | 31.77684 |  |  |
| InPy | 460.1659 | 77.35173 | 122.2157 |  | 97.24217 |
| BghiP | 436.2094 |  | 42.84931 |  |  |
| **TOTAL** | **3132.037** | **1271.894** | **1556.819** | **1168.273** | **1307.596** |
|  |  |  |  |  |  |
| 2 Ring |  | 132.8935 | 168.9297 | 145.8916 | 187.6999 |
| 3 Ring | 1085.339 | 917.9695 | 1007.31 | 903.7953 | 869.0563 |
| 4 Ring | 407.4878 | 44.45459 | 60.28762 | 21.56632 | 57.65243 |
| 5 Ring | 742.8347 | 99.22451 | 155.2263 | 97.02028 | 95.94536 |
| 6 Ring | 896.3754 | 77.35173 | 165.065 |  | 97.24217 |
| LMW | 1085.339 | 1050.863 | 1176.24 | 1049.687 | 1056.756 |
| HMW | 2046.698 | 221.0308 | 380.5789 | 118.5866 | 250.84 |
| cPAHs | 2046.698 | 221.0308 | 380.5789 | 118.5866 | 250.84 |
| LMW/HMW | 0.530288 | 4.754373 | 3.09066 | 8.851648 | 4.21287 |
|  |  |  |  |  |  |
| Phe/Ant |  |  |  |  |  |
| Fla/Pyr |  |  |  |  |  |
| An/Ant+Phe | |  |  |  |  |
| Fla/Fla+Pry | |  |  |  |  |
| Chry/BaA | 1.297439 |  | 0.663667 |  | 0.94435 |
| An/178 |  |  |  |  |  |
| BaA/228 | 0.777922 | 0.194976 | 0.158938 | 0.094589 | 0.130049 |
| BaA/BaA+Chry | 0.435267 | 1 | 0.601082 | 1 | 0.514311 |
| InPy/InPy+BghiP | 0.513363 | 1 | 0.740409 |  | 1 |

|  |  |  |  |  |  |
| --- | --- | --- | --- | --- | --- |
|  | **JUNE** |  |  |  |  |
|  | **A1** | **A2** | **A3** | **A4** | **A5** |
| Nap | 540.7738 | 335.1806 | 170.5708 | 161.157 |  |
| Ace |  | 42.84994 |  |  |  |
| Acy | 153.3918 | 13.37553 |  |  |  |
| Flu | 340.6786 |  | 782.5638 |  | 619.3865 |
| Ant | 15.86054 |  | 28.82055 |  |  |
| Phe |  |  |  |  |  |
| Flt | 365.1388 |  |  |  |  |
| Pyr | 47.42627 |  |  | 565.348 |  |
| BaA | 196.4116 | 86.00726 | 44.23267 | 344.3517 |  |
| Chry | 38.69357 | 840.5823 | 55.42074 | 901.6221 | 583.9984 |
| BbF | 1.798752 | 182.3355 | 38.02563 | 28.48815 | 26.52454 |
| BkF | 18.36941 | 183.7896 | 97.73961 | 1020.772 | 157.619 |
| BaP | 227.2366 | 1095.404 | 99.36249 |  | 66.28815 |
| DiahA | 139.9134 | 114.2485 | 169.2665 | 1114.129 |  |
| InPy | 52.44128 | 235.6395 | 104.7031 | 1098.762 |  |
| BghiP | 153.7859 | 1569.288 | 40.29143 | 5234.63 |  |
| **TOTAL** | **2291.92** | **4698.701** | **1630.997** | **10469.26** | **1453.817** |
|  |  |  |  |  |  |
| 2 Ring | 540.7738 | 335.1806 | 170.5708 | 161.157 |  |
| 3 Ring | 509.931 | 56.22547 | 811.3844 |  | 619.3865 |
| 4 Ring | 647.6702 | 926.5895 | 99.6534 | 1811.322 | 583.9984 |
| 5 Ring | 387.3181 | 1575.778 | 404.3943 | 2163.39 | 250.4317 |
| 6 Ring | 206.2271 | 1804.928 | 144.9945 | 6333.392 |  |
| LMW | 1050.705 | 391.4061 | 981.9552 | 161.157 | 619.3865 |
| HMW | 1241.215 | 4307.295 | 649.0422 | 10308.1 | 834.43 |
| cPAHs | 828.6504 | 4307.295 | 649.0422 | 9742.755 | 834.43 |
| LMW/HMW | 0.846513 | 0.090871 | 1.51293 | 0.015634 | 0.742287 |
|  |  |  |  |  |  |
| Phe/Ant |  |  |  |  |  |
| Fla/Pyr | 7.699082 |  |  |  |  |
| An/Ant+Phe | 1 |  |  |  |  |
| Fla/Fla+Pry | 0.885045 |  |  |  |  |
| Chry/BaA | 0.197002 | 9.773388 | 1.252937 | 2.618318 |  |
| An/178 | 0.089104 |  | 0.161913 |  |  |
| BaA/228 | 0.861455 | 0.377225 | 0.194003 | 1.510314 |  |
| BaA/BaA+Chry | 0.83542 | 0.092821 | 0.443865 | 0.276372 |  |
| InPy/InPy+BghiP | 0.254289 | 0.130553 | 0.722118 | 0.173487 |  |

|  |  |  |  |  |  |
| --- | --- | --- | --- | --- | --- |
|  |  |  |  |  |  |
|  | **MIN** | **MAX** | **AVERAGE** | **SD** | **SE** |
| Nap | 132.8935338 | 540.7738 | 234.6544 | 131.9314 | 26.38628 |
| Ace | 21.19469 | 73.2093 | 50.64891 | 24.54792 | 4.909585 |
| Acy | 0.140904361 | 263.7116 | 59.29127 | 75.41347 | 15.08269 |
| Flu | 25.521 | 1085.339 | 540.3418 | 386.1178 | 77.22355 |
| Ant | 15.8605429 | 902.154 | 333.7446 | 292.147 | 58.42939 |
| Phe | 24.12405 | 238.5176 | 97.67764 | 66.07918 | 13.21584 |
| Flt | 5.730600221 | 643.428 | 411.2219 | 287.1581 | 57.43161 |
| Pyr | 21.7074 | 565.348 | 108.2314 | 131.888 | 26.37761 |
| BaA | 21.56632174 | 1099.761 | 250.7804 | 319.6425 | 63.9285 |
| Chry | 24.04982609 | 901.6221 | 256.5979 | 236.9119 | 47.38238 |
| BbF | 1.798751996 | 804.2827 | 186.9823 | 204.0149 | 40.80298 |
| BkF | 12.8104766 | 1020.772 | 197.5417 | 295.4199 | 59.08399 |
| BaP | 34 | 1095.404 | 198.6192 | 248.5335 | 49.7067 |
| DiahA | 31.77684348 | 1114.129 | 307.7281 | 390.354 | 78.07079 |
| InPy | 52.44128078 | 1098.762 | 267.7757 | 278.2497 | 55.64993 |
| BghiP | 40.29142591 | 5234.63 | 884.5361 | 1500.696 | 300.1392 |
|  | **1168.273487** | **10469.26** | **4386.373** | **1852.226** | **973.821** |
